# Two glycoside hydrolases decompose the sporangium matrix to release spores during sporangium dehiscence in *Actinoplanes missouriensis*

**DOI:** 10.1128/mbio.02682-25

**Published:** 2025-11-04

**Authors:** Kyota Mitsuyama, Shixuan Hu, Naoki Sunagawa, Kiyohiko Igarashi, Takeaki Tezuka, Yasuo Ohnishi

**Affiliations:** 1 Department of Biotechnology, Graduate School of Agricultural and Life Sciences, The University of Tokyo 13143, Bunkyo-ku, Tokyo, Japan; 2 Department of Biomaterial Sciences, Graduate School of Agricultural and Life Sciences, The University of Tokyo 13143, Bunkyo-ku, Tokyo, Japan; 3 Collaborative Research Institute for Innovative Microbiology, The University of Tokyo 13143, Bunkyo-ku, Tokyo, Japan; University of California Berkeley, Berkeley, California, USA

**Keywords:** sporangium dehiscence, spore release, glycoside hydrolase, zoospore

## Abstract

The actinomycete *Actinoplanes missouriensis* grows as a substrate mycelium and forms terminal sporangia containing a few hundred spores surrounded by an unidentified matrix called the sporangium matrix. Upon contact with water, sporangia release spores through a process called sporangium dehiscence, in which the opaque sporangium envelope becomes transparent, and sporangia swell with water, followed by spore release via the breakage of the outer envelope. In this study, we identified two paralogous hydrolases (GimA and GimB) required for sporangium dehiscence. Transcription of *gimA* and *gimB* is activated during sporangium dehiscence by a global transcriptional activator, TcrA. GimA and GimB are composed of an N-terminal signal peptide, glycoside hydrolase domain, and galactose-binding-like domain. No phenotypic changes were observed between the wild-type strain and single-gene null mutants of *gimA* and *gimB*. The double mutant (Δ*gimA*Δ*gimB* strain) formed normal sporangia, but spores were not released under dehiscence-inducing conditions; the sporangium envelope became transparent, but spores remained in the sporangium matrix. The release of spores from Δ*gimA*Δ*gimB* sporangia was immediately induced by the exogenous addition of recombinant GimA or GimB. Thin-layer chromatography revealed that GimA and GimB cleaved the substrate polysaccharide at different positions. Null mutants of genes within a putative polysaccharide biosynthesis gene cluster adjacent to *gimB* generated distorted sporangia, and no sporangium matrix was produced in one of the mutants. We conclude that a major component of the sporangium matrix is a polysaccharide consisting of repeating oligosaccharides and that GimA and GimB hydrolyze this polysaccharide to release spores during sporangium dehiscence.

**IMPORTANCE:**

Dispersal by means of zoospores is a common feature in the life cycle of many lower eukaryotes, including chytrid fungi, oomycetes, and some bacteria of actinomycetes. However, the molecular mechanisms underlying zoospore release from (zoo)sporangia remain largely unknown. Here, we revealed that two paralogous glycoside hydrolases (GimA and GimB) play essential roles in the release of spores, which can swim in aquatic environments as zoospores, from sporangia in *Actinoplanes missouriensis*, a filamentous soil-inhabiting bacterium. During sporangium dehiscence, GimA and GimB are produced and secreted extracellularly to hydrolyze the polysaccharide component of the sporangium matrix that encapsulates the spores. This study clarifies an unprecedented molecular mechanism in the process of zoospore release from the sporangia.

## INTRODUCTION

A sporangium is an enclosed structure in which spores are formed. It is produced by eukaryotes (moss and fern plants [1], fungi [2], and some stramenopiles [2, 3]) and bacteria (4, 5) at some stage of their life cycle. Some sporangia of eukaryotes, such as chytrid fungi and oomycetes, are referred to as zoosporangia because they contain zoospores (2, 3). Some bacterial sporangia of actinomycetes, such as *Actinoplanes*, *Catenuloplanes*, *Dactylosporangium*, *Kineosporia*, *Planomonospora*, and *Spirillospora*, contain spores that can swim as zoospores after being released from sporangia (6–8). (Zoo)sporangia release zoospores (or spores that become zoospores) in aqueous environments in response to certain signals. However, the molecular mechanisms of zoospore release in both eukaryotes and bacteria are largely unknown. In particular, the structural components of bacterial sporangia remain to be characterized, and the molecular basis of their degradation is unknown; bacterial sporangia have no exit in contrast to the sporangia of chytrid fungi, and the outer envelope and intrasporangial matrix must be decomposed to release spores.

Members of the genus *Actinoplanes* were the first sporangium-forming gram-positive bacteria to be discovered (9–11). They are soil-dwelling actinomycetes and are characterized by complex morphological development. *Actinoplanes missouriensis* is the model species of this genus (12). It forms a branched substrate mycelium during vegetative growth and subsequently produces globose or subglobose terminal sporangia that grow from the substrate mycelium via short sporangiophores on humic acid-trace element (HAT) agar (13). Each sporangium contains a few hundred spores encased within a membranous substance (spore sheath) (6), and the confined space among spores inside a sporangium is filled with an intrasporangial matrix called the sporangium matrix (4). In response to water exposure, sporangia open and release spores via a process referred to as sporangium dehiscence. After release from sporangia, the spore sheath is removed to disperse free zoospores, which begin swimming in aquatic environments using flagella and exhibit chemotactic properties toward various substances, including sugars, amino acids, and aromatic compounds (6, 8, 14, 15). In a niche suitable for vegetative growth, zoospores stop swimming and begin to germinate (8). On HAT agar, small sporangium-like structures are observed after 2 or 3 days of cultivation at 30°C (13). Then, mature sporangia that can release spores under sporangium dehiscence-inducing conditions are formed after 5–7 days of cultivation (16, 17).

In the elaborate life cycle of *A. missouriensis*, sporangium dehiscence is the initial stage of the awakening of dormant spores (17). Sporangium dehiscence can be induced by suspending sporangia in 25 mM histidine solution for 1 h. According to time-course observation by phase-contrast microscopy, the entire process of sporangium dehiscence can be divided into three stages as follows. (i) Immediately after suspension in 25 mM histidine solution, sporangia appeared phase-bright (see Fig. 2A as an example). (ii) Then, the sporangium envelope became transparent, and sporangia swelled with water (see Fig. 2B as an example). (iii) Finally, spores were released from sporangia and started swimming using flagella as free zoospores. The sporangium envelope is composed of three layers of different thicknesses (18, 19). We assume that the sporangium envelope degraded, leaving its skeletal structure, when it became transparent. Therefore, this time-course observation suggests the possibility that the sporangium matrix, in addition to the sporangium envelope, plays a pivotal role in preventing spore release because spores were not released for a short period after the sporangium envelope became transparent at stage (ii).

In this study, we focused on two genes, *AMIS_23960* and *AMIS_66390*, the transcription of which was shown to be highly upregulated during sporangium dehiscence in our previous RNA-Seq analysis (17) (accession number DRA012687 in the DDBJ Sequence Read Archive), to elucidate the molecular mechanisms underlying sporangium dehiscence in *A. missouriensis*. These gene products share high amino acid sequence identity and are predicted to function as glycoside hydrolases. Here, we named AMIS_23960 GimA and AMIS_66390 GimB (glycosidase required for the hydrolysis of intrasporangial matrix) for their functions revealed in this study. Here, we describe the functional characterization of GimA and GimB, which demonstrated that these glycoside hydrolases are specifically required for the release of spores during sporangium dehiscence. Our study also provides important insights into the component of the sporangium matrix, which is an unexplored constituent of the sporangium.

## RESULTS

### TcrA activates the transcription of *gimA* and *gimB*, which encode secreted glycoside hydrolase paralogs, during sporangium dehiscence

The *gimA* and *gimB* genes encode proteins of 498 and 521 amino acids, respectively, which share 62% amino acid sequence identity (Fig. S1A). The SignalP 6.0 server (https://services.healthtech.dtu.dk/services/SignalP-6.0/) predicted that GimA and GimB have a signal peptide for the general secretory (Sec) pathway (the predicted cleavage sites are between residues 30 and 31 and residues 28 and 29, respectively). A protein database search using InterPro version 93.0 (https://www.ebi.ac.uk/interpro/) showed that the N-terminal portions of mature GimA and GimB harbor a family 5 glycoside hydrolase domain (accession number IPR001547; residues 69–300 and residues 91–298, respectively). In addition, a galactose-binding-like domain is located in the C-terminal portions of GimA and GimB (IPR008979; residues 348–495 and residues 371–519, respectively) (Fig. S1A). According to the carbohydrate-active enzymes database (CAZy; http://www.cazy.org), GimA and GimB belong to the GH5 enzyme family, which is one of the largest glycoside hydrolase families. These *in silico* analyses suggest that GimA and GimB hydrolyze the glycosidic bonds between two or more sugars.

**Fig 1:**
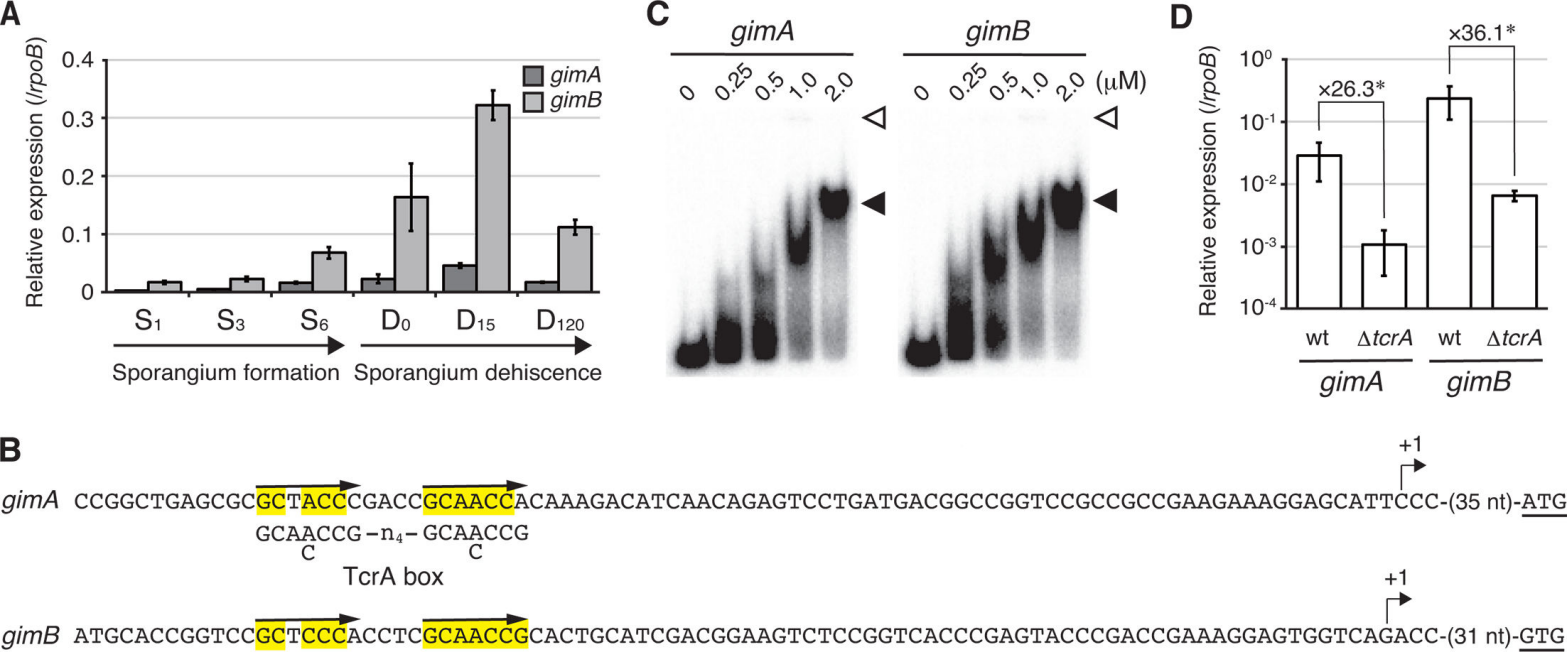
Transcription of *gimA* and *gimB* is regulated by TcrA. (**A**) Transcript levels of *gimA* and *gimB*. The transcripts were examined by qRT-PCR analysis. RNA samples were prepared from substrate hyphae or mixtures of substrate hyphae and sporangia grown on HAT agar for 1, 3, and 6 days (S_1_, S_3_, and S_6_, respectively) and sporangia (including some substrate hyphae) incubated in 25 mM histidine solution to induce sporangium dehiscence for 0, 15, and 120 min (D_0_, D_15_, and D_120_, respectively). The *rpoB* transcript, encoding the RNA polymerase β subunit, was used as an internal standard. Data represent mean values from three biological replicates ± standard errors. (**B**) Nucleotide sequences of the regions upstream from *gimA* and *gimB*. The transcriptional start points are indicated by bent arrows. Putative TcrA boxes (composed of a direct repeat sequence) are indicated by arrows, and consensus TcrA box sequences are shown below the sequences. The translational start codons are underlined. (**C**) Binding of polyhistidine-tagged TcrA (His-TcrA) to the *gimA*- and *gimB*-promoter-containing regions. The DNA fragments were ^32^P-labeled and used as probes in electrophoretic mobility shift assays. The amount of His-TcrA used is shown above the lanes. The positions of the wells and His-TcrA-bound probes are shown by open and closed triangles, respectively. (**D**) Transcript levels of *gimA* and *gimB* in the wild-type (wt) and Δ*tcrA* strains. Transcripts were examined by qRT-PCR using RNA samples extracted from the mixture of sporangia and mycelia of both strains incubated in 25 mM histidine solution for 15 min. The *rpoB* transcript was used as the internal standard. Data are the mean values from three biological replicates ± standard errors. Differences are analyzed by the Student’s *t*-test, and the level of significance is *P < 0.05.

We analyzed the transcript levels of *gimA* and *gimB* in the wild-type strain using quantitative reverse transcription PCR (qRT-PCR). Transcripts were detected at relatively low levels in the substrate hyphae or a mixture of substrate hyphae and sporangia cultivated on HAT agar at 30°C for 1, 3, and 6 days (Fig. 1A). We then induced sporangium dehiscence by suspending a mixture of substrate hyphae and sporangia in 25 mM histidine solution, followed by incubation at room temperature for 120 min. Under these conditions, the transcript levels of *gimA* and *gimB* markedly increased during sporangium dehiscence and decreased after the completion of sporangium dehiscence (Fig. 1A). These data indicate that *gimA* and *gimB* transcription is activated during sporangium dehiscence.

The transcriptional start points of *gimA* and *gimB* were determined to be 38 and 35 nucleotides upstream from the start codons, respectively, by high-resolution S1 nuclease mapping (Fig. S2). A global transcriptional regulator, TcrA, controls sporangium formation, spore dormancy, and sporangium dehiscence by binding to a 21 bp direct repeat sequence (TcrA box), 5′-nnGCA(A/C)CCG-n_4_-GCA(A/C)CCGn-3′. TcrA specifically binds to the TcrA box to activate transcription in its regulon (4). Notably, putative TcrA boxes were found in regions upstream from the transcriptional start sites of *gimA* and *gimB* (Fig. 1B). These locations, which are typical of the TcrA box, suggested that TcrA directly activates the transcription of *gimA* and *gimB*. Consistent with this notion, we showed that a recombinant polyhistidine-tagged TcrA protein bound to the regions upstream from *gimA* and *gimB* in electrophoretic mobility shift assays (EMSAs; Fig. 1C). Furthermore, we quantified the transcript levels of *gimA* and *gimB* in the wild-type and *tcrA* null mutant (Δ*tcrA*) strains by qRT-PCR using RNA samples extracted from a mixture of sporangia and substrate hyphae incubated in 25 mM histidine solution at room temperature for 15 min. We found that the transcript levels of *gimA* and *gimB* in the Δ*tcrA* strain were much lower than those in the wild-type strain (26- and 36-fold, respectively; Fig. 1D), indicating that TcrA directly activates the transcription of these genes. It should be noted that neither *gimA* nor *gimB* was identified as a TcrA regulon in our previous study, in which the transcriptomes of the wild-type and Δ*tcrA* strains were analyzed using RNA samples extracted from the cells cultivated on HAT agar at 30°C for 6 days; the amount of *gimA* and *gimB* transcripts did not change significantly (1.7- and 1.2-fold, respectively, downregulated in the Δ*tcrA* strain compared to the wild-type strain) (4). The transcriptomes of the wild-type and Δ*tcrA* strains were analyzed only at the late stage of sporangium formation, and those during sporangium dehiscence were not investigated in our previous study. This result also supports our proposal that GimA and GimB function during sporangium dehiscence.

### A double mutant of *gimA* and *gimB* fails to release spores from sporangia

To examine the *in vivo* functions of GimA and GimB, we generated null mutant strains of *gimA* (Δ*gimA*) and *gimB* (Δ*gimB*) by deleting most of their coding sequences from the wild-type strain. No phenotypic change was observed among the wild-type, Δ*gimA*, and Δ*gimB* strains in macroscopic observation of mycelia and sporangia formed on nutrient-rich yeast extract-beef extract-NZ amine-maltose monohydrate (YBNM) agar and nutrient-poor HAT agar (data not shown). Thus, we observed mycelia and sporangia grown on HAT agar using scanning electron microscopy (SEM), but the Δ*gimA* and Δ*gimB* strains produced normal sporangia with short sporangiophores (Fig. S3). Furthermore, phase-contrast microscopy revealed that sporangia of the Δ*gimA* and Δ*gimB* strains opened normally and released spores under dehiscence-inducing conditions (Fig. S4). Consistent with this observation, sporangia of the Δ*gimA* and Δ*gimB* strains released a similar number of spores as the wild-type sporangia (Fig. S5). Overall, these observations indicated that the deletion of *gimA* or *gimB* had no effect on mycelial growth, sporangium formation, and sporangium dehiscence under the tested conditions.

**Fig 2:**
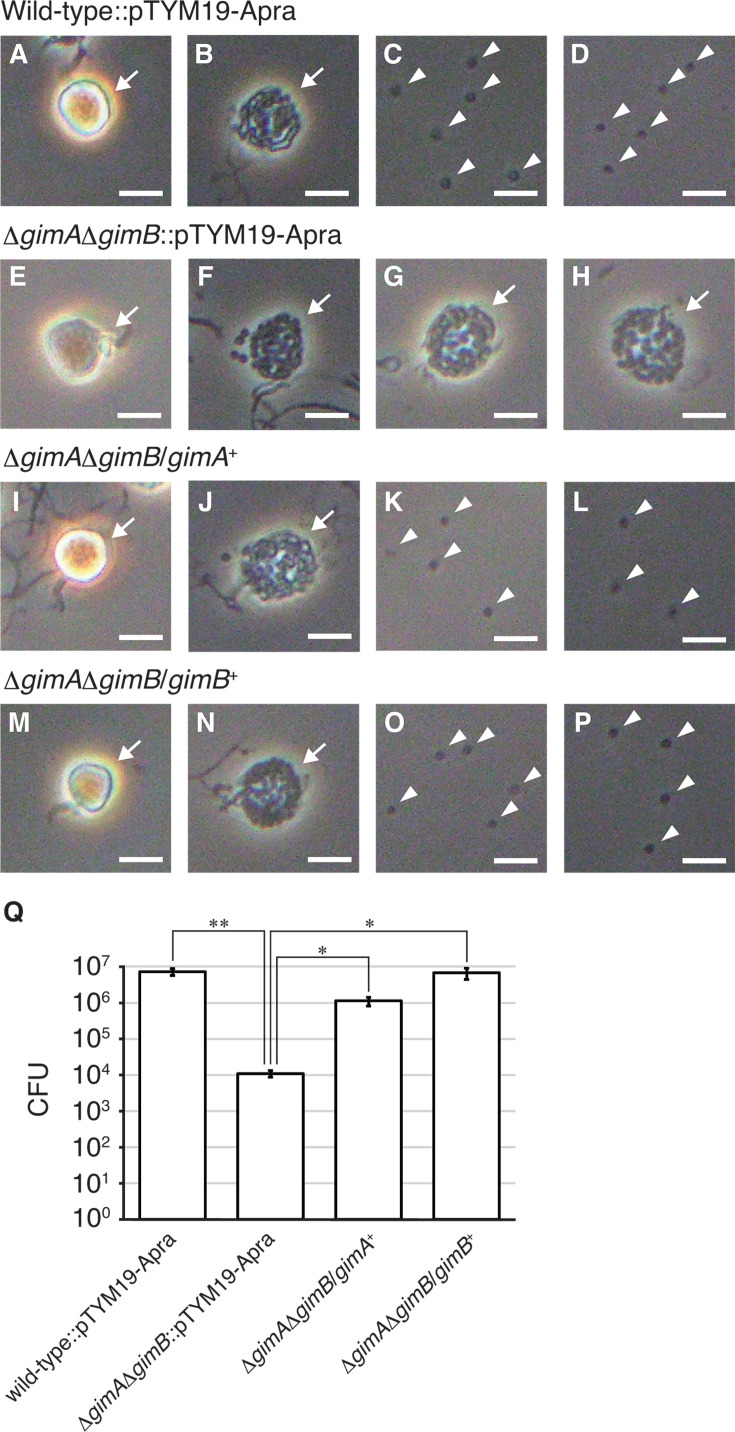
Observation of sporangium dehiscence and number of spores released from sporangia. (**A–P**) Observation of sporangia and zoospores using phase-contrast microscopy. Sporangia produced on HAT agar were harvested and suspended in 25 mM histidine solution to induce sporangium dehiscence. Micrographs of the wild-type strain harboring pTYM19-Apra (**A–D**), Δ*gimA*Δ*gimB* strain harboring pTYM19-Apra (**E–H**), Δ*gimA*Δ*gimB* strain harboring the *gimA* complementation plasmid (**I–L**), and Δ*gimA*Δ*gimB* strain harboring the *gimB* complementation plasmid (**M–P**) are shown. Panels A, E, I, and M show images taken immediately after suspension. Panels B, F, J, and N show images taken 15 min after suspension. Panels C, G, K, and O show images taken 30 min after suspension. Panels D, H, L, and P show images taken 60 min after suspension. Immediately after suspension, sporangia appeared phase-bright (**A, E, I, and M**). The sporangium envelope gradually became transparent before the release of spores (**B, J, and N**). In the Δ*gimA*Δ*gimB* strain harboring pTYM19-Apra, spore release was not observed, although the sporangium envelope became transparent (**F, G, and H**). Sporangia and released spores are indicated by arrows and arrowheads, respectively. Bars, 5 μm. (**Q**) Number of spores released from sporangia. Each strain was cultivated on HAT agar at 30°C for 7 days. Zoospores released from sporangia by pouring 25 mM NH_4_HCO_3_ solution on the sporangium-forming agar were counted as colony-forming units (CFUs) on YBNM agar. The values represent the mean ± standard error of three biological replicates. Differences are analyzed by the Student’s *t*-test, and the levels of significance are *P < 0.05 and **P < 0.01.

Next, considering the possibility that GimA and GimB exert similar physiological functions, we generated a double mutant of *gimA* and *gimB* (Δ*gimA*Δ*gimB*) by deleting most of the coding sequence of *gimB* from the Δ*gimA* strain. No macroscopic difference was observed in the mycelia or sporangia formed on YBNM and HAT agar between the wild-type and Δ*gimA*Δ*gimB* strains (data not shown). We observed mycelia and sporangia of the Δ*gimA*Δ*gimB* strain grown on HAT agar using SEM, but the mutant formed normal sporangia similar to those of the wild-type strain (Fig. S3). Thus, we analyzed sporangium dehiscence in the wild-type and Δ*gimA*Δ*gimB* strains using phase-contrast microscopy. In this experiment, a chromosome-integrating vector, pTYM19-Apra (5, 20), was introduced into both strains for the gene complementation test described below. Under sporangium dehiscence-inducing conditions using 25 mM histidine solution, wild-type sporangia normally opened and released zoospores (Fig. 2A through D). In contrast, the sporangium envelope of the Δ*gimA*Δ*gimB* strain became transparent, but almost no spores were released into the aquatic environment, indicating that the gene products of *gimA* and *gimB* are involved in spore release from sporangia (Fig. 2E through H). Because sporangium dehiscence was completed in the Δ*gimA* and Δ*gimB* strains, as described above (Fig. S4), spore release seemed to proceed normally in the presence of either GimA or GimB. To support this, we quantified the spores released from sporangia of the wild-type and Δ*gimA*Δ*gimB* strains, both of which contained pTYM19-Apra, by counting the colonies formed on YBNM agar after incubation at 30°C for 2 days. While the wild-type sporangia formed on one HAT agar plate released approximately 10^7^ spores, Δ*gimA*Δ*gimB* sporangia released only ~10^4^ spores per HAT agar plate (Fig. 2Q), which is consistent with the observation by phase-contrast microscopy (Fig. 2A through H). For the gene complementation test, *gimA* and *gimB*, with their native promoters, were individually introduced into the Δ*gimA*Δ*gimB* strain using pTYM19-Apra. We found that the sporangia of the complementation strains opened normally and released spores under dehiscence-inducing conditions (Fig. 2I through P). Furthermore, the number of spores released from sporangia was restored by the introduction of *gimA* or *gimB*; sporangia of the Δ*gimA*Δ*gimB* strains harboring *gimA* or *gimB* formed on one HAT agar plate released approximately 10^6^ or 10^7^ spores, respectively (Fig. 2Q). These results clearly demonstrate that the production of either GimA or GimB provides the Δ*gimA*Δ*gimB* strain with the ability to release spores from the sporangia.

### Exogenous addition of recombinant GimA or GimB protein induces spore release from Δ*gimA*Δ*gimB* sporangia

**Fig 3:**
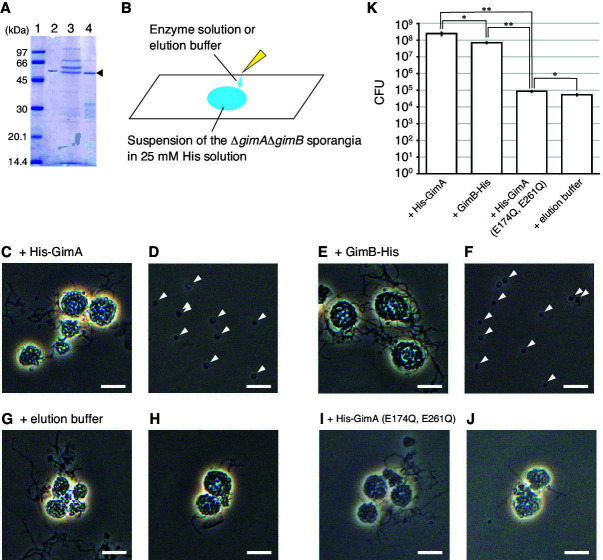
Spore release induced by recombinant GimA and GimB proteins. (**A**) SDS-PAGE of affinity-purified proteins. Lane 1, molecular weight markers; lane 2, His-GimA protein; lane 3, GimB-His protein; lane 4, His-GimA (E174Q, E261Q) variant. The recombinant proteins are indicated by the arrowhead. (**B**) Schematic representation of the experimental procedure. Sporangia of the Δ*gimA*Δ*gimB* strain were suspended in 25 mM histidine solution to induce sporangium dehiscence and transferred to a glass slide. Solutions containing recombinant proteins or elution buffer were added to the suspension immediately before observation by phase-contrast microscopy. (**C–J**) Observation of spore release from sporangia using phase-contrast microscopy. His-GimA protein (**C and D**), GimB-His protein (**E and F**), elution buffer (**G and H**), or His-GimA (E174Q, E261Q) protein (**I and J**) was added to the suspension of sporangia. Panels C, E, G, and I were obtained immediately after addition. Panels D, F, H, and J were taken 15 min after addition. In panels D and F, spores are indicated by arrowheads. Bars, 10 μm. (**K**) Number of released spores induced by recombinant proteins. NH_4_HCO_3_ solution (25 mM) was poured on the sporangium-forming agar plates of the Δ*gimA*Δ*gimB* strain to induce sporangium dehiscence, followed by the addition of the protein solution of His-GimA, GimB-His, or His-GimA (E174Q, E261Q) or elution buffer. Spores released from sporangia were counted as colony-forming units (CFUs) on YBNM agar. The values represent the mean ± standard error of three biological replicates. Differences are analyzed by the Student’s *t*-test, and the levels of significance are *P < 0.05 and **P < 0.01.

We produced recombinant GimA and GimB proteins with polyhistidine tags at the N- and C-terminal ends (His-GimA and GimB-His), respectively, in *Escherichia coli* and purified them (Fig. 3A). The sporangium matrix-hydrolyzing activity of His-GimA and GimB-His was examined as follows: (i) Δ*gimA*Δ*gimB* sporangia and mycelia harvested from the sporangium-forming HAT agar surface were suspended in 25 mM histidine solution; (ii) the suspension was incubated at room temperature until the sporangium envelope of most sporangia became transparent when observed by phase-contrast microscopy; (iii) the suspension was divided into three equal parts; (iv) His-GimA, GimB-His, or an elution buffer used for the purification of recombinant proteins (negative control) was added to each part; and (v) the suspensions were observed by phase-contrast microscopy (Fig. 3B). We found that spores were released from the Δ*gimA*Δ*gimB* sporangia immediately after the addition of His-GimA or GimB-His proteins, whereas almost no spores were released in the suspension with elution buffer (Fig. 3C through H; Movie S1). These results demonstrate that GimA and GimB hydrolyze the sporangium matrix to release the spores.

In most glycoside hydrolases, the hydrolysis of glycosidic bonds is catalyzed by two amino acid residues via the classical Koshland mechanism: a general acid (proton donor) and a nucleophile/base. For family 5 glycoside hydrolases, both the catalytic proton donor and nucleophile/base are glutamate residues. For an endocellulase E1 from *Acidothermus cellulolyticus*, these catalytic residues were shown to be Glu_162 and Glu_282 (21–24). Amino acid sequence alignment between the glycoside hydrolase domains of GimA and endocellulase E1 from *A. cellulolyticus* suggested that Glu_174 and Glu_261 function as catalytic residues in GimA (Fig. S1B). Thus, we generated mutations within the *gimA* gene, which replaced these glutamate residues with glutamine in the gene product. A GimA variant with a polyhistidine tag at the N-terminal end (His-GimA [E174Q, E261Q]) was produced in *E. coli* and purified (Fig. 3A). We examined the sporangium matrix-hydrolyzing activity of His-GimA (E174Q, E261Q) using the method described above. As expected, almost no spores were released from the Δ*gimA*Δ*gimB* sporangia in the suspension containing His-GimA (E174Q, E261Q) (Fig. 3I and J). This result demonstrates that the catalytic activity of the glycoside hydrolase domain in GimA is essential for the hydrolysis of the sporangium matrix.

Next, the sporangium matrix-hydrolyzing activity of the exogenously added proteins was quantitatively examined as follows. (i) Ten milliliters of 25 mM NH_4_HCO_3_ solution was poured on each of the sporangium-forming HAT agar plates of the Δ*gimA*Δ*gimB* strain, followed by incubation at room temperature for 1 h. (ii) The protein solution containing His-GimA, GimB-His, or His-GimA (E174Q, E261Q) and the elution buffer were added to the solution on the agar plates, followed by incubation at room temperature for 1 h. (iii) The spore-containing solution was collected from the agar plate and filtered through a 5 μm membrane filter to eliminate hyphae and sporangia. (iv) A portion of the filtered solution was inoculated onto YBNM agar, followed by incubation at 30°C for 2 days. The number of spores released from sporangia was estimated from the number of colonies formed on YBNM agar. We found that His-GimA and GimB-His induced the release of approximately 10^8^ and 10^7^ spores, respectively (Fig. 3K), which is consistent with the observation by phase-contrast microscopy (Fig. 3C through F). Because His-GimA induced the release of a larger number of spores than GimB-His in this experiment, His-GimA seems to have higher activity than GimB-His. Meanwhile, exogenous addition of His-GimA (E174Q, E261Q) protein induced a significantly lower number of spores than His-GimA and GimB-His (Fig. 3K), which is also consistent with the observation by phase-contrast microscopy (Fig. 3I and J). The number of spores released by the mutant protein was slightly higher than that released by the elution buffer alone (Fig. 3K). This may be due to the residual activity of the His-GimA (E174Q, E261Q) protein, the presence of the protein itself, or contaminating proteins in the protein solution.

### Detection of the products of sporangium matrix hydrolysis by GimA and GimB

**Fig 4:**
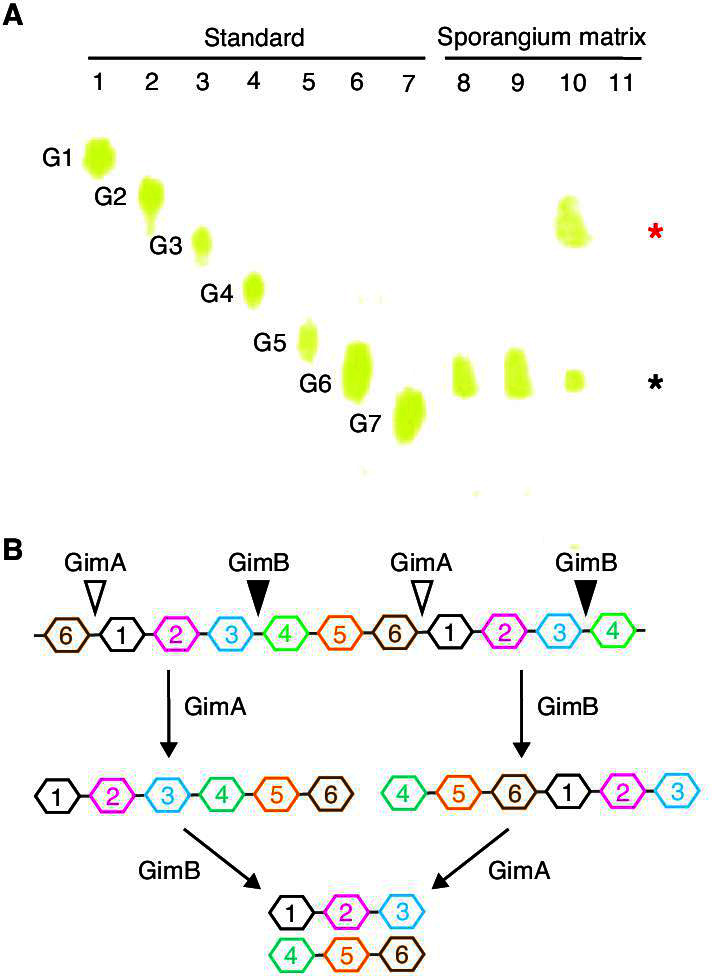
Hydrolysis of sporangium matrix by recombinant GimA and GimB proteins. (**A**) Thin-layer chromatography of sporangium matrix hydrolysis products. After sporangia of the Δ*gimA*Δ*gimB* strain were suspended and incubated in 25 mM histidine solution, recombinant His-GimA, GimB-His, or both proteins were added to the suspension of sporangia to induce the hydrolysis of the sporangium matrix. Elution buffer was used as the negative control. After incubation at room temperature, the reaction products were collected via ethanol precipitation and subjected to TLC. The TLC plate was developed using isopropanol/ethanol/water (7:1:2, vol/vol/vol). Sugars were detected with 3% (vol/vol) H_2_SO_4_ in ethanol containing 6.5 mM *p*-hydroxybenzhydrazide. Lanes 1–7, standards; glucose (G1), cellobiose (G2), cellotriose (G3), cellotetraose (G4), cellopentaose (G5), cellohexaose (G6), and celloheptaose (G7) were used. Lanes 8–10, products of sporangium matrix hydrolysis by His-GimA (lane 8), GimB-His (lane 9), and both proteins (lane 10). Lane 11, negative control reaction using elution buffer. The black asterisk on the right side of the panel indicates the reaction product generated by His-GimA or GimB-His. The red asterisk indicates the product generated by the combined action of both proteins. (**B**) Schematic representation of a hypothetical model of sporangium matrix hydrolysis by GimA and GimB, assuming that repeating oligosaccharides are hexasaccharides. As described in the main text, repeating oligosaccharides are likely to be between four and eight sugars. Open and closed triangles show glycosidic linkages hydrolyzed by GimA and GimB, respectively. In this hypothetical model, hydrolysis by either GimA or GimB generates hexasaccharides, whereas hydrolysis by both proteins produces trisaccharides.

The experimental results described above indicate that a polysaccharide is an important structural component of the sporangium matrix. We prepared sporangia of the Δ*gimA*Δ*gimB* strain with a transparent sporangium envelope by suspending and incubating the sporangia in 25 mM histidine solution and divided the suspension of sporangia into four equal parts. Then, His-GimA, GimB-His, both His-GimA and GimB-His, and the elution buffer were individually added to each of the four parts. After the release of spores from sporangia was confirmed by phase-contrast microscopy in the suspensions with His-GimA, GimB-His, and both His-GimA and GimB-His, the products of sporangium matrix hydrolysis were isolated by ethanol precipitation (see Materials and Methods). Thin-layer chromatography (TLC) revealed that oligosaccharides of a certain length were generated in the suspensions with either His-GimA or GimB-His (Fig. 4A, lanes 8 and 9). The mobility of this oligosaccharide on TLC was similar to that of a hexasaccharide comprising glucose. However, it cannot be concluded that this oligosaccharide is composed of hexasaccharides because the constituent sugars of this oligosaccharide are unknown. Considering that the mobility of oligosaccharides on TLC changes as the constituent sugars change, this oligosaccharide is considered to contain between four and eight sugars. Interestingly, oligosaccharides of approximately half the length (the mobility on TLC was similar to that of the trisaccharide consisting of glucose) were also detected in the suspension containing both His-GimA and GimB-His (Fig. 4A, lane 10). In contrast, no sugars were detected in the suspension with elution buffer using the same experimental procedure (Fig. 4A, lane 11), indicating that these oligosaccharides of two different lengths are the products of enzymatic hydrolysis of the sporangium matrix by His-GimA and/or GimB-His. These results strongly indicate that a major component of the sporangium matrix is a polysaccharide consisting of repeating oligosaccharides (four to eight sugars), and that GimA and GimB cleave the polysaccharide at different positions in the oligosaccharide unit; the cutting positions of both are two to four sugars apart (Fig. 4B).

### A gene cluster adjacent to *gimB* is involved in sporangium matrix production

**Fig 5:**
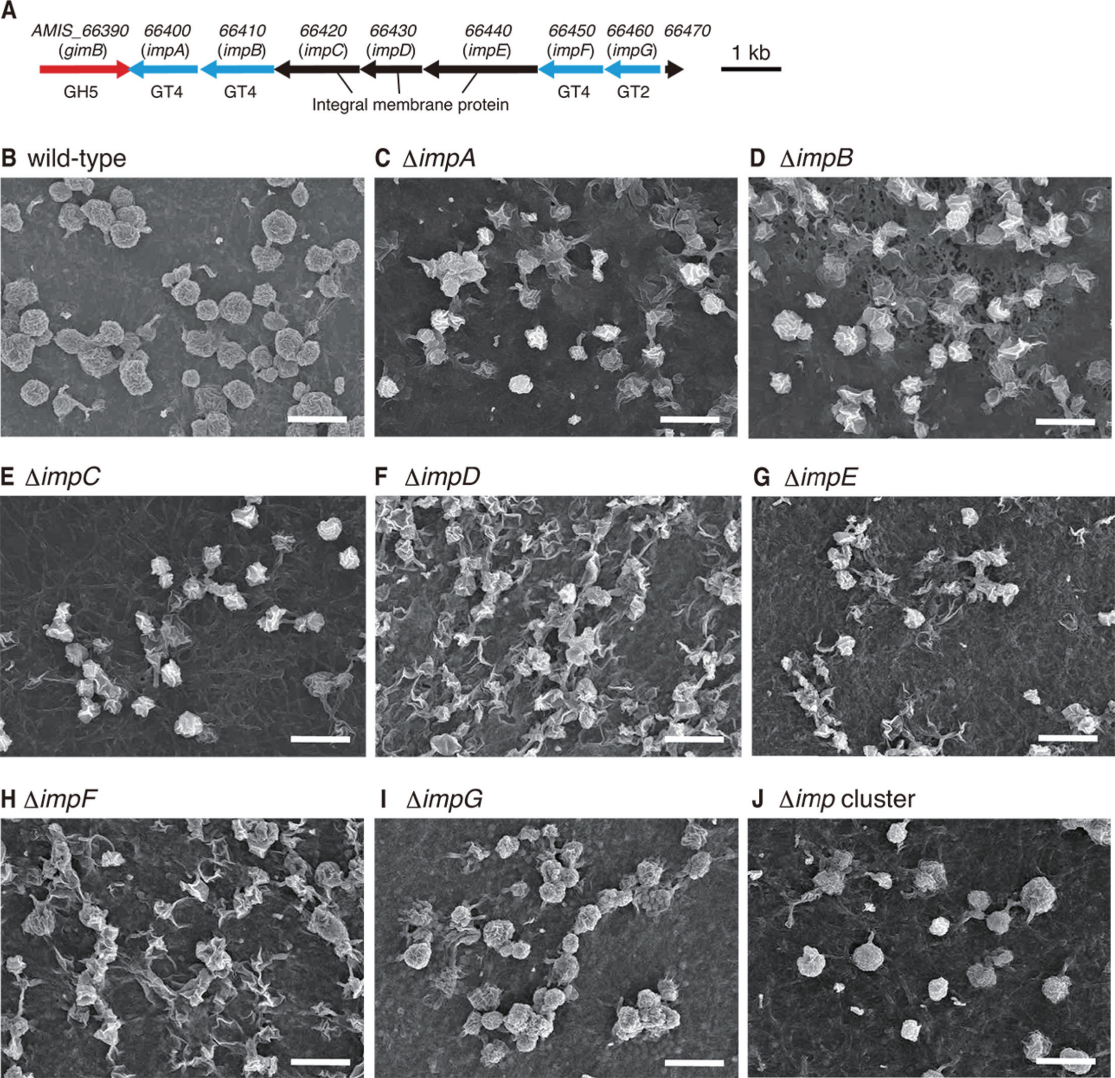
Gene organization of the *imp* gene cluster and SEM observation of mycelia and sporangia formed on HAT agar after 7 days of cultivation. (**A**) Gene organization of the *imp* gene cluster. Arrows indicate the locations of the open reading frames, including their length and direction. Gene identification numbers are shown above the arrows. Gene names are shown in parentheses. GH5, glycoside hydrolase family 5; GT4, glycosyltransferase family 4; GT2, glycosyltransferase family 2. (**B**) Wild-type strain. (**C**) Δ*impA* strain. (**D**) Δ*impB* strain. (**E**) Δ*impC* strain. (**F**) Δ*impD* strain. (**G**) Δ*impE* strain. (**H**) Δ*impF* strain. (**I**) Δ*impG* strain. (**J**) Δ*imp* cluster strain. Bars, 10 μm.

We focused on a gene cluster comprising seven genes, *AMIS_66400–AMIS_66460*, located adjacent to *gimB* on the *A. missouriensis* chromosome (Fig. 5A; Fig. S6A). Homologs of the gene products are widely conserved among the genus *Actinoplanes*, which share 51%–90% amino acid sequence identity with the proteins of *A. missouriensis*. According to our previous RNA-Seq analysis, transcription of the gene cluster was activated at the early stage of sporangium formation (3 days of cultivation on HAT agar) in the wild-type strain (Fig. S6C). Notably, the transcript levels of the genes within the gene cluster were not significantly different between the wild-type and Δ*tcrA* strains at the late stage of sporangium formation (6 days of cultivation on HAT agar; 1.1- to 3.0-fold upregulated in the Δ*tcrA* strain) (4). We named AMIS_66400–AMIS_66460 ImpA–ImpG (intrasporangial matrix polysaccharide biosynthesis) for the functions described below. A protein database search using InterPro revealed that the N-terminal portions of ImpA, ImpB, and ImpF harbor a glycosyltransferase family 4 domain (IPR028098; residues 10–178, 14–206, and 14–175, respectively). In addition, the C-terminal portions of these proteins harbor a glycosyltransferase group 1 domain (IPR001296 or PF13692; residues 201–336, 238–376, and 190–329, respectively). ImpG harbors a glycosyltransferase family 2 domain (IPR001173; residues 65–188). ImpC harbors a polysaccharide biosynthesis and transport domain (IPR050833; residues 13–418). A protein database search using the Conserved Domain Database (https://www.ncbi.nlm.nih.gov/Structure/cdd/wrpsb.cgi) identified a domain of the Alg6_Alg8 superfamily in ImpE (cl46505; residues 163–461). Meanwhile, no domains were predicted in ImpD (Fig. S6B). According to the CAZy database, ImpA, ImpB, and ImpF belong to the GT4 enzyme family, whereas ImpG belongs to the GT2 family. The TMHMM 2.0 server (https://services.healthtech.dtu.dk/services/TMHMM-2.0/) predicted that ImpC, ImpD, and ImpE contain 11, 5, and 11 transmembrane helices, respectively (Fig. S6B). Overall, the gene cluster encodes four glycosyltransferases and three integral membrane proteins.

To examine the *in vivo* functions of the gene cluster, we generated null mutant strains of each of the seven genes (Δ*impA*–Δ*impG* strains) and the whole gene cluster (Δ*imp* cluster strain). We observed mycelia and sporangia in the wild-type and mutant strains grown on HAT agar at 30°C for 7 days using SEM. The wild-type strain produced normal globose or subglobose sporangia with short sporangiophores (Fig. 5B). In contrast, all single-gene mutant strains produced sporangia in squashed shapes, indicating that sporangium formation did not proceed appropriately in these strains (Fig. 5C through I). In the Δ*impG* strain, normal globose or subglobose sporangia were observed, in addition to abnormal sporangia (Fig. 5I). The Δ*imp* cluster strain produced irregular sporangia with abnormal shapes, although normal sporangia were also observed (Fig. 5J).

**Fig 6:**
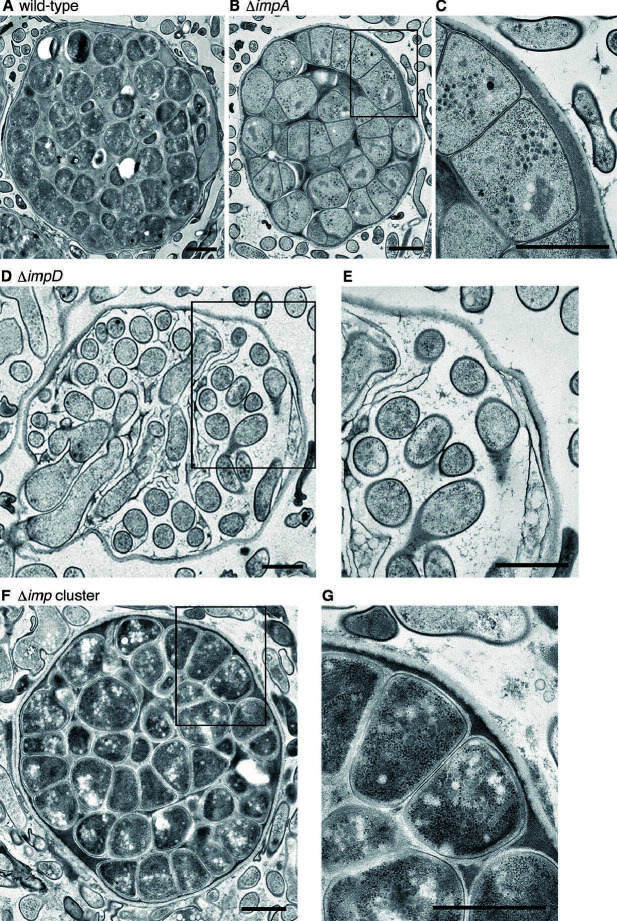
TEM analysis of ultrathin sections of sporangia produced on HAT agar. (**A**) Wild-type strain. (**B and C**) Δ*impA* strain. (**D and E**) Δ*impD* strain. (**F and G**) Δ*imp* cluster strain. Panels C, E, and G are enlarged views of a part of the images shown in panels B, D, and F, respectively. Bars, 1 μm.

We performed transmission electron microscopy (TEM) analysis of the sporangia of the Δ*impA*, Δ*impD*, and Δ*imp* cluster strains grown under the same conditions as for SEM analysis. As described in our previous study (25), the wild-type strain produced mature round spores of similar size surrounded by a sporangium matrix (Fig. 6A). In contrast, spores in the Δ*impA* sporangia were not fully mature, and hyphae that underwent septation were observed (Fig. 6B and C). In the Δ*impD* strain, the space among spores inside the sporangium was larger than that in the wild-type strain, and no sporangium matrix seemed to be produced (Fig. 6D and E). When suspended in water, the Δ*impD* sporangia were phase-dark immediately after suspension, but no spores were observed inside the sporangia by phase-contrast microscopy (Fig. S7). Therefore, the cells of various sizes observed in the Δ*impD* sporangia seem to be extending mycelia, not spores (Fig. 6D), suggesting that the sporangium matrix is also important for spore formation inside sporangia. In the Δ*imp* cluster strain, round spores of similar size were observed (Fig. 6F), which was consistent with the SEM analysis (Fig. 5J). However, the cytoplasm of spores and the space among spores inside the sporangium appeared dark in the Δ*imp* cluster strain, suggesting the possibility that an unknown substance accumulated (Fig. 6G).

**Fig 7:**
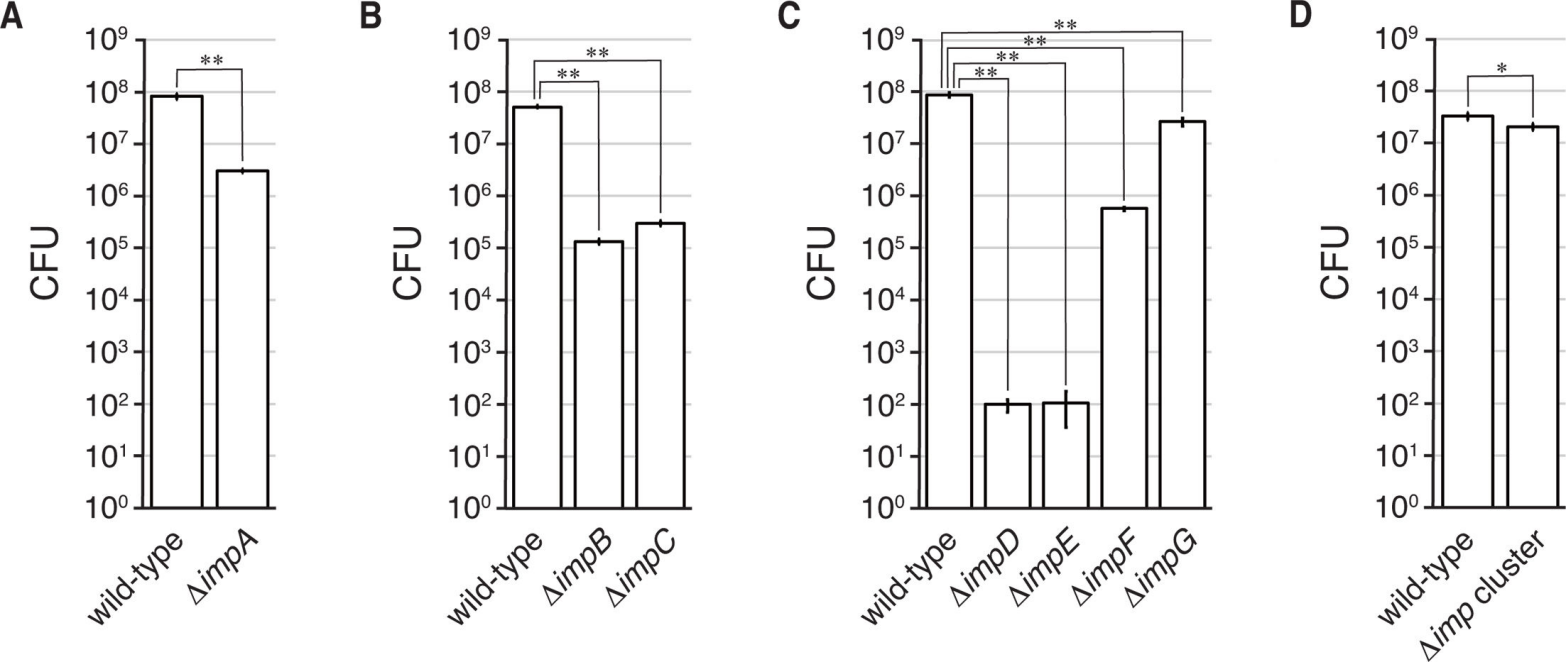
Number of spores released from sporangia. Zoospores released from sporangia by pouring 25 mM NH_4_HCO_3_ solution were counted as colony-forming units (CFUs) on YBNM agar. Data are mean values ± standard errors from at least three biological replicates. Differences are analyzed by the Student’s *t*-test, and the levels of significance are *P < 0.05 and **P < 0.01. In panels **A–D**, the wild-type strain was used as a control.

We quantified the spores released from sporangia of the wild-type and mutant strains by counting the colonies formed on YBNM agar after incubation at 30°C for 2 days. Under the conditions used in this assay, the number of spores released from the sporangia of the Δ*impA*, Δ*impB*, Δ*impC*, Δ*impD*, Δ*impE*, and Δ*impF* strains was lower than that from the wild-type sporangia (Fig. 7A through C). Among the mutants, the sporangia of Δ*impD* and Δ*impE* released only 10^2^ spores (Fig. 7C). Meanwhile, sporangia of the Δ*impG* and Δ*imp* cluster strains released a similar number of spores as the wild-type sporangia (Fig. 7C and D). These results clearly demonstrate that this gene cluster is involved in sporangium matrix biosynthesis during sporangium formation.

## DISCUSSION

In the present study, we found that GimA and GimB play a pivotal role in sporangium dehiscence in *A. missouriensis*. These proteins are predominantly produced during sporangium dehiscence and are presumably secreted into the sporangium matrix space via the general secretion pathway, where they function as glycoside hydrolases to decompose sporangium matrix polysaccharides to release spores into the external environment. To the best of our knowledge, this is the first report describing the components of the bacterial sporangium matrix and its degrading enzymes, as well as a molecular mechanism for spore release from bacterial sporangium. In *A. missouriensis*, the global transcriptional activator TcrA regulates genes required for sporangium formation, spore dormancy, and sporangium dehiscence (4). Because *gimA* and *gimB* were shown to be new members of the TcrA regulon (Fig. 1B through D), this study also expanded the TcrA regulatory network, which is deeply involved in the morphological development of *A. missouriensis* (Fig. S8).

Phase-contrast microscopy revealed that sporangium dehiscence in *A. missouriensis* proceeds through three consecutive stages (Fig. 2A through C). The sporangium envelope seems to be decomposed during the transition from the first to second stages of sporangium dehiscence because an obvious change in the surface layers was observed by phase-contrast microscopy (Fig. 2A and B). This assumption was supported by the observation that sporangia somewhat expanded in size during the second stage, which implied that the sporangium matrix swelled with water from the external environment (Fig. 2B). In this study, we observed that Δ*gimA*Δ*gimB* sporangia were not able to release spores due to the failure of sporangium matrix degradation. This observation clearly demonstrates that the sporangium matrix, in addition to the sporangium envelope, functions as a primary component that prevents the release of spores from sporangia (Fig. 2B). Furthermore, the experimental results in this study provide significant insights into the structural elements of the sporangium matrix. Exogenous addition of recombinant His-GimA or GimB-His proteins to the Δ*gimA*Δ*gimB* sporangia, whose dehiscence process proceeded until the sporangium envelope became transparent, immediately induced spore release (Fig. 3C through F). Therefore, the sporangium matrix is inevitably the target of hydrolysis by these glycoside hydrolases, and major constituents of the sporangium matrix are predicted to be polysaccharides. To the best of our knowledge, GimA and GimB are the first enzymes to hydrolyze matrix components inside sporangia (or zoosporangia) formed by microorganisms. We expect that these polysaccharides, which should be produced and secreted into the sporangium matrix space during sporangium formation, shield spores inside a sporangium from various environmental stresses, such as heat and desiccation, until they are hydrolyzed during sporangium dehiscence.

What is the composition of sporangium matrix polysaccharides? The substrate specificities of GimA and GimB may provide insight into this question. Both enzymes belong to family 5 glycoside hydrolases. A variety of reaction and substrate specificities are known for the enzymes in this family, such as endo- and exoglucanase, endo- and exomannanase, β-glucosidase, and β-mannosidase. As shown in the CAZy database, most enzymes in this family have been assigned to 55 subfamilies based on their sequences and associated biochemical data (26). However, GimA and GimB have not yet been classified into subfamilies. Thus, we constructed a phylogenetic tree using 467 glycoside hydrolases from family 5, including GimA and GimB. GimA and GimB were found to be in a clade containing several functionally characterized β-mannosidases (Fig. S9): *Bl*GH5_18 and *Bl*Man5B from the gut bacterium *Bifidobacterium longum* depolymerize the 2-acetamido-2-deoxy-4-*O*-(β-D-mannopyranosyl)-D-glucopyranose (Man-β-1-4-Glc*N*Ac) disaccharide, which comprises the universal core of eukaryotic *N*-glycans (27, 28), whereas Caci_2257 from *Catenulispora acidiphila* has a specific activity against the synthetic substrate *p*-nitrophenyl-β-D-mannopyranoside (29). This result suggests that GimA and GimB may also have β-mannosidase activity. We incubated each of the following seven polysaccharides as a substrate candidate with His-GimA: (i) gum guar (β-D-1,4-Man [main chain] and highly branched Gal [side chain]), (ii) gum arabic (β-D-1,3-Gal [main chain] and β-D-1,6-Gal [side chain]), (iii) locust bean gum (β-D-1,4-Man [main chain] and Gal [side chain]), (iv) glucomannan, (v) mannan from *Saccharomyces cerevisiae* (β-1,4-mannose [main chain]), (vi) laminarin (β-1,3-1,6-glucose [main chain]), and (vii) curdlan (β-1,3-glucose [main chain]). However, no product was detected by TLC in any of the reaction mixtures. Meanwhile, we added α-mannosidase to a suspension of Δ*gimA*Δ*gimB* sporangia with a transparent sporangium envelope, which had been incubated in 25 mM histidine solution. However, almost no spores were released from the sporangia when observed using phase-contrast microscopy. Thus, no information was obtained from these trials regarding the substrate specificity of GimA and the composition of sporangium matrix polysaccharides. However, we assume that sporangium matrix polysaccharides should be novel polysaccharides. In contrast, the analysis of the products of sporangium matrix hydrolysis by GimA and GimB provided significant information about these issues. TLC analysis of the products generated by the hydrolysis of the sporangium matrix clearly demonstrated that GimA and GimB have different substrate specificities (Fig. 4A). Based on this result, we propose a working model for the hydrolysis of the sporangium matrix: GimA and GimB hydrolyze different glycosidic linkages of the repeating units in polysaccharides (Fig. 4B).

We identified the *imp* gene cluster as a promising candidate gene cluster for sporangium matrix biosynthesis. As described in the Results section, ImpC harbors a polysaccharide biosynthesis and transport domain. Several members harboring this domain function as floppases that facilitate the movement of lipid-linked oligosaccharide precursors from the inner to the outer leaflet of the cytoplasmic membrane (30). In bacteria, the Wzx/Wzy-dependent pathway produces extracellular polysaccharides. In this pathway, oligosaccharide repeating units are assembled by several glycosyltransferases inside the cell and translocated across the cytoplasmic membrane by a floppase (Wzx protein). Polymerization of repeating units occurs by the Wzy protein on the outer leaflet of the cytoplasmic membrane (30). Therefore, we expect that the gene products of the *imp* gene cluster provide a Wzx/Wzy-dependent pathway, in which ImpC functions as a Wzx protein. Considering that ImpE is predicted to harbor the Alg6_Alg8 superfamily domain (31), which catalyzes glycosyltransferase reactions, ImpE may function as a Wzy protein. Members of the GT4 family are anomer-retaining enzymes that catalyze glycosyltransferase reactions using NDP-sugars as donors. GT4 family glycosyltransferases have a GT-B fold, in which N- and C-terminal domains are generally responsible for binding to sugar acceptors and NDP-sugars, respectively (32). Thus, we constructed a phylogenetic tree using ImpA, ImpB, ImpF, and 86 functionally characterized glycosyltransferases from the GT4 family. ImpA, ImpB, and ImpF were found to be in a clade containing mannosyltransferases, *N*-acetylglucosaminyltransferases, and glucosyltransferases (Fig. S10A). We also constructed a phylogenetic tree using ImpG and 94 functionally characterized glycosyltransferases from the GT2 family. ImpG was found to be in a clade containing a glucosyltransferase, *N*-acetylglucosaminyltransferase, *N*-acetylgalactosaminyltransferase, formylarabinosyltransferase, and mannosyltransferase (Fig. S10B). These phylogenetic analyses suggest that a repeat unit of the sporangium matrix polysaccharide is a heterooligosaccharide, whose sugar moieties may be composed of mannose, *N*-acetylglucosamine, glucose, *N*-acetylgalactosamine, and/or formylarabinose.

Exogenous addition of either His-GimA or GimB-His induced the release of spores from Δ*gimA*Δ*gimB* sporangia (Fig. 3), indicating that GimA and GimB share redundant physiological functions in the release of spores from sporangia. This is supported by the fact that no phenotypic changes were observed among the wild-type, Δ*gimA*, and Δ*gimB* strains (Fig. S3 to S5). His-GimA appears to have a higher sporangium matrix-hydrolyzing activity than GimB-His, as a larger number of spores were released from the Δ*gimA*Δ*gimB* sporangia by the exogenous addition of His-GimA than of GimB-His (Fig. 3K). In the gene complementation test, however, a larger number of spores were released from the sporangia of the Δ*gimA*Δ*gimB* strain harboring the *gimB* complementation plasmid than from the Δ*gimA*Δ*gimB* strain harboring the *gimA* complementation plasmid (Fig. 2Q). Considering that the transcript levels of *gimB* were much higher than those of *gimA* in the wild-type strain (Fig. 1A), we assume that the amount of GimB produced in the Δ*gimA*Δ*gimB* strain harboring *gimB* was larger than that of GimA produced in the Δ*gimA*Δ*gimB* strain harboring *gimA*. GimA/GimB homologs are widely conserved in *Actinoplanes* species, which share 47%–80% amino acid sequence identity with GimA. Furthermore, proteins showing high similarity to GimA/GimB are also conserved in other actinomycete species, including *Paractinoplanes toevensis* and *Symbioplanes lichenis*. Members of the genera *Paractinoplanes* and *Symbioplanes* are closely related to *Actinoplanes* (33). In contrast, homologs were not found in members of the genera *Micromonospora*, *Catenuloplanes*, *Couchioplanes*, *Dactylosporangium*, or *Pilimelia*, all of which belong to the family *Micromonosporaceae*, together with the genus *Actinoplanes*. Interestingly, the species harboring the GimA/GimB homologs usually contain two to four copies of these glycoside hydrolases, suggesting that multiple copies of paralogs provide certain advantages from an evolutionary viewpoint. We expect that these paralogous glycoside hydrolases have different substrate specificities, similar to those of GimA and GimB. Because of the different cleavage sites in the repeating unit, the production of both GimA and GimB proteins, rather than either GimA or GimB, is likely to contribute to the efficient hydrolysis of sporangium matrix components. Future investigations of GimA and GimB will provide further clues to the molecular mechanisms of sporangium dehiscence and the chemical structures of sporangium matrix polysaccharides.

## MATERIALS AND METHODS

### General methods

Bacterial strains, plasmid vectors, and media used in this study have been described previously (5, 34, 35). Primers used in this study are listed in Table S1. *A. missouriensis* cells were prepared as previously described (4). SEM was performed using an S-4800 scanning electron microscope (Hitachi, Tokyo, Japan) as described previously (36). The ultrathin sections of sporangia were observed with a JEM-1400Plus electron microscope (Jeol, Tokyo, Japan) by Tokai Electron Microscopy, Inc. (Aichi, Japan). Phase-contrast microscopic observations of sporangia and zoospores were performed using a BH-2 microscope (Olympus, Tokyo, Japan), as described previously (37). Free zoospores were quantified as previously described (38).

### RNA extraction

*A. missouriensis* cells were disrupted by grinding with a mortar and pestle, and the cell lysate was mixed with a lysis/binding solution from the RNAqueous Total RNA Isolation Kit (Thermo Fisher Scientific, MA, USA). After debris was removed by centrifugation at 21,000 × *g* for 5 min, total RNA was extracted according to the manufacturer’s instructions. Total RNA was treated with DNase I to eliminate contaminating genomic DNA and purified by phenol-chloroform extraction and ethanol precipitation.

### qRT-PCR

Total RNA samples (1 μg each) were used for reverse transcription for first-strand cDNA synthesis using the ThermoScript RT-PCR system (Thermo Fisher Scientific) according to the manufacturer’s instructions. Following RNase H treatment, the synthesized cDNA libraries were used as PCR templates. Quantitative PCR was performed using the SYBR premix *Ex Taq* II reaction mixture (Takara Biochemicals, Shiga, Japan) and the AriaMx Real-Time PCR System (Agilent Technologies, CA, USA) under the following conditions: 5 min at 95°C, followed by 40 cycles of 5 s at 95°C and 10 s at 60°C. The *rpoB* gene (*AMIS_5940*) was used as the internal standard. All reactions were performed in biological triplicate, and the data were normalized using the average of the internal standard.

### S1 nuclease mapping

S1 nuclease mapping was performed as previously described (39, 40). Hybridization probes were prepared using PCR and labeled at both 5′-ends with [γ-^32^P]ATP (220 TBq/mmol; PerkinElmer, MA, USA) using T4 polynucleotide kinase (Takara Biochemicals). Labeling at one side of the 5′-ends was eliminated by digestion with *Eco*RI. For each hybridization, 40 μg of total RNA was used. Protected fragments were analyzed on 6% polyacrylamide DNA sequencing gels according to a previously described method (41).

### EMSAs

DNA fragments containing the *gimA* or *gimB* promoter regions were prepared by PCR and ^32^P-labeled at the 5′-ends using [γ-^32^P]ATP (220 TBq/mmol; PerkinElmer) and T4 polynucleotide kinase (Takara Biochemicals). Recombinant His-TcrA protein was incubated with the labeled DNA probes at 25°C for 15 min in binding buffer [25 mM NaH_2_PO_4_, 7.5% glycerol, 100 ng/μL bovine serum albumin, and 25 ng/μL poly(dI-dC)], and the mixtures were subjected to 6% native PAGE at 34 mA for 2 h. Recombinant His-TcrA protein was prepared as described previously (4).

### Construction of gene deletion mutants

To construct the gene deletion mutant strains, the upstream and downstream regions of the target genes were amplified by PCR. The amplified fragments were digested with *Eco*RI and *Xba*I (for upstream regions) or *Xba*I and *Hin*dIII (for downstream regions) and cloned into pUC19 digested with the same restriction enzymes. The generated plasmids were sequenced to confirm the absence of errors. The cloned fragments were digested with *Eco*RI and *Xba*I (for upstream regions) or *Xba*I and *Hin*dIII (for downstream regions) and cloned together into pK19mob*sacB* (42), whose kanamycin resistance gene had been replaced with the apramycin resistance gene *aac(3)IV*, digested with *Eco*RI and *Hin*dIII. The generated plasmids were introduced into *A. missouriensis* by conjugation as described previously (43). Apramycin-resistant colonies generated by single crossover recombination were isolated. One of them was cultivated in peptone-yeast extract-magnesium liquid broth at 30°C for 48 h, and the mycelia suspended in 0.75% NaCl solution were spread onto Czapek-Dox broth (BD, NJ, USA) agar medium containing extra sucrose (final concentration 5%). After incubation at 30°C for 5 days, sucrose-resistant colonies were inoculated onto YBNM agar medium with or without apramycin to confirm that they were sensitive to apramycin. Apramycin-sensitive and sucrose-resistant colonies generated by the second crossover recombination were isolated as candidates for gene deletion mutants. Disruption of the target genes was confirmed by PCR (data not shown). To construct the Δ*gimA*Δ*gimB* strain, *gimB* was disrupted using the Δ*gimA* strain as the parental strain. The Δ*tcrA* strain had been generated in the previous study (4).

### Construction of strains for gene complementation testing

The 2.4- and 2.7-kbp DNA fragments containing the promoter and coding sequences of *gimA* and *gimB*, respectively, were amplified by PCR. The amplified fragments were digested with *Eco*RI and *Hin*dIII and cloned into pTYM19-Apra (4, 20) digested with the same restriction enzymes. The generated plasmids were sequenced to confirm that no PCR-derived error was present. These plasmids were introduced into the Δ*gimA*Δ*gimB* strain by conjugation as described previously (44). An empty plasmid pTYM19-Apra was also introduced into the wild-type and Δ*gimA*Δ*gimB* strains for the vector control strains. Apramycin-resistant colonies were obtained.

### Preparation of recombinant GimA and GimB proteins

The 1.4- and 1.5-kbp DNA fragments containing the *gimA*- and *gimB*-coding sequences, respectively, without those for signal peptides were amplified by PCR. The fragments were digested with *Eco*RI and *Hin*dIII and cloned into pUC19 digested with the same restriction enzymes. The generated plasmids were sequenced to confirm that no PCR-derived error was introduced. For *gimA*, the cloned fragment was digested with *Nde*I and *Hin*dIII and cloned into pColdII digested with the same restriction enzymes. For *gimB*, the cloned fragment was digested with *Nde*I and *Hin*dIII and cloned into pET26b digested with the same restriction enzymes. The plasmids were then introduced into *E. coli* BL21 (DE3) cells. For His-GimA production, transformants were cultivated in LB broth (100 mL) at 37°C for 2.5 h and at 15°C for 30 min. Then, isopropyl β-D-1-thiogalactopyranoside (IPTG) was added to the culture at a final concentration of 0.1 mM, followed by further cultivation at 15°C for 30 h. For GimB-His production, transformants were cultivated in LB broth (100 mL) at 37°C for 2.5 h. Then, IPTG was added to the culture at a final concentration of 0.1 mM, followed by further cultivation at 30°C for 5 h. The *E. coli* cells were collected by centrifugation at 3,000 × *g* for 10 min, suspended in 5 mL of lysis buffer (50 mM NaH_2_PO_4_, 300 mM NaCl, 10 mM imidazole, and 10% glycerol, pH 8.0), and disrupted by sonication. After the removal of cell debris by centrifugation at 10,000 × *g* for 30 min, His-GimA and GimB-His proteins were purified from cell-free extracts using Ni-nitrilotriacetic acid Superflow resin (Qiagen, Tokyo, Japan) according to the manufacturer’s instructions. His-GimA and GimB-His were eluted using elution buffer (50 mM NaH_2_PO_4_, 300 mM NaCl, 400 mM imidazole, and 10% glycerol, pH 8.0). For the His-GimA (E174Q, E261Q) protein, the mutated DNA fragment was amplified using overlap extension PCR. The mutant protein was produced and purified according to the method described for His-GimA. The quality of the purified proteins was assessed using SDS-PAGE. The concentration of the protein solution was adjusted to a similar signal intensity of each protein on the SDS-PAGE gel. Purification of recombinant His-TcrA protein was performed as described previously (4).

### Observation of GimA- and GimB-induced spore release and counting of released spores

Sporangia and mycelia of the Δ*gimA*Δ*gimB* strain were harvested from the sporangium-forming HAT agar surface using a spatula and suspended in 25 mM histidine solution. The suspension was incubated with rotation at room temperature for 1 h to induce sporangium dehiscence. Next, 5 μL of the suspension was transferred to a glass slide. After 1 μL of each recombinant protein solution or elution buffer was mixed with the suspension on a glass slide, the mixture was observed under a BH-2 phase-contrast microscope. To quantify the released spores, 10 mL of 25 mM NH_4_HCO_3_ solution was poured to each sporangium-forming HAT agar plate of the Δ*gimA*Δ*gimB* strain, followed by incubation at room temperature for 1 h. Then, 80 μL of each protein solution or elution buffer was added to the solution on the agar plate, followed by incubation at room temperature for 1 h. After being collected from the plate, the spore suspension was filtered through a 5-μm Acrodisc membrane filter (Pall Corporation, NY, USA) to eliminate hyphae and sporangia. A portion of the filtrate was inoculated on YBNM agar, and the plate was incubated at 30°C for 2 days. From the number of colonies formed on YBNM agar, the number of spores released from sporangia was estimated.

### Hydrolysis of sporangium matrix by GimA and GimB

Sporangia of the Δ*gimA*Δ*gimB* strain with a transparent sporangium envelope were prepared using 25 mM histidine solution as described above. Sporangia were collected by centrifugation and suspended in 20 mM Tris-HCl (pH 7.4) buffer containing 150 mM NaCl. The suspension of sporangia was divided into four equal parts. Then, (i) His-GimA protein, (ii) GimB-His protein, (iii) both proteins, or (iv) elution buffer was added to each part. After incubation at room temperature for 1 h, we confirmed that almost all sporangia incubated with (i) His-GimA, (ii) GimB-His, or (iii) both proteins released spores, using phase-contrast microscopy. Proteinase K was then added to each suspension, followed by incubation at room temperature for 3 h. The suspensions were centrifuged at 21,000 × *g* for 10 min at 4°C, and the supernatant was collected. Then, twice the amount of ethanol was added to the supernatant. After the mixture was incubated at −20°C for 1 h, the mixture was centrifuged at 21,000 × *g* for 10 min at 4°C, and the supernatant was eliminated to isolate the precipitate. The precipitate was dissolved in distilled water, and the solution was centrifuged at 21,000 × *g* for 10 min at 4°C. The supernatant was then retrieved and freeze-dried.

### TLC

The isolated products of sporangium matrix hydrolysis were dissolved in distilled water and subjected to TLC. Glucose, cellobiose, cellotriose, cellotetraose, cellopentaose, cellohexaose, and celloheptaose were used as sugar standards (glucose, cellobiose, cellotriose, and cellotetraose, 100 μg each/lane; cellopentaose, cellohexaose, and celloheptaose, 200 μg each/lane). The plate was developed with isopropanol/ethanol/water (7:1:2, vol/vol/vol) and detected by heat treatment after soaking the plate in freshly prepared 3% H_2_SO_4_ in methanol containing 6.5 mM *p*-hydroxybenzhydrazide.

## SUPPLEMENTAL MATERIAL

Supplemental material mbio.02682-25-s0001.pdf Figures S1 to S10, Table S1, legend for Movie S1, and supplemental reference.

Movie S1 mbio.02682-25-s0002.mov Induction of spore release from Δ*gimA*Δ*gimB* sporangia by His-GimA protein.

ASM does not own the copyrights to Supplemental Material that may be linked to, or accessed through, an article. The authors have granted ASM a non-exclusive, world-wide license to publish the Supplemental Material files. Please contact the corresponding author directly for reuse.
