# Supplementary material for: Two glycoside hydrolases decompose the sporangium matrix to release spores during sporangium dehiscence in Actinoplanes missouriensis
Source: mBio. 2025 Nov 4;16(12):e02682-25. doi: 10.1128/mbio.02682-25 (PMC12691653; doi:10.1128/mbio.02682-25)
Supplement: Supplemental material — Figures S1 to S10, Table S1, legend for Movie S1, and supplemental reference. [file mbio.02682-25-s0001.pdf]

Supplemental material for

Two glycoside hydrolases decompose sporangium matrix to release spores during sporangium dehiscence in *Actinoplanes missouriensis*

Kyota Mitsuyama,[1] Shixuan Hu,[1] Naoki Sunagawa,[2,3] Kiyohiko Igarashi,[2,3] Takeaki Tezuka,[1,3,*] Yasuo Ohnishi[1,3,*]

[1]Department of Biotechnology, Graduate School of Agricultural and Life Sciences, The University of Tokyo, Bunkyo-ku, Tokyo, Japan

[2]Department of Biomaterial Sciences, Graduate School of Agricultural and Life Sciences, The University of Tokyo, Bunkyo-ku, Tokyo, Japan

[3]Collaborative Research Institute for Innovative Microbiology, The University of Tokyo, Bunkyo-ku, Tokyo, Japan

*Address correspondence to Takeaki Tezuka, atezuka@mail.ecc.u-tokyo.ac.jp; Yasuo Ohnishi, ayasuo@mail.ecc.u-tokyo.ac.jp

This PDF file includes:

Figures S1 to S10
Table S1
Legend for Movie S1
Supplemental reference

Other supporting material for this manuscript includes the following:

Movie S1

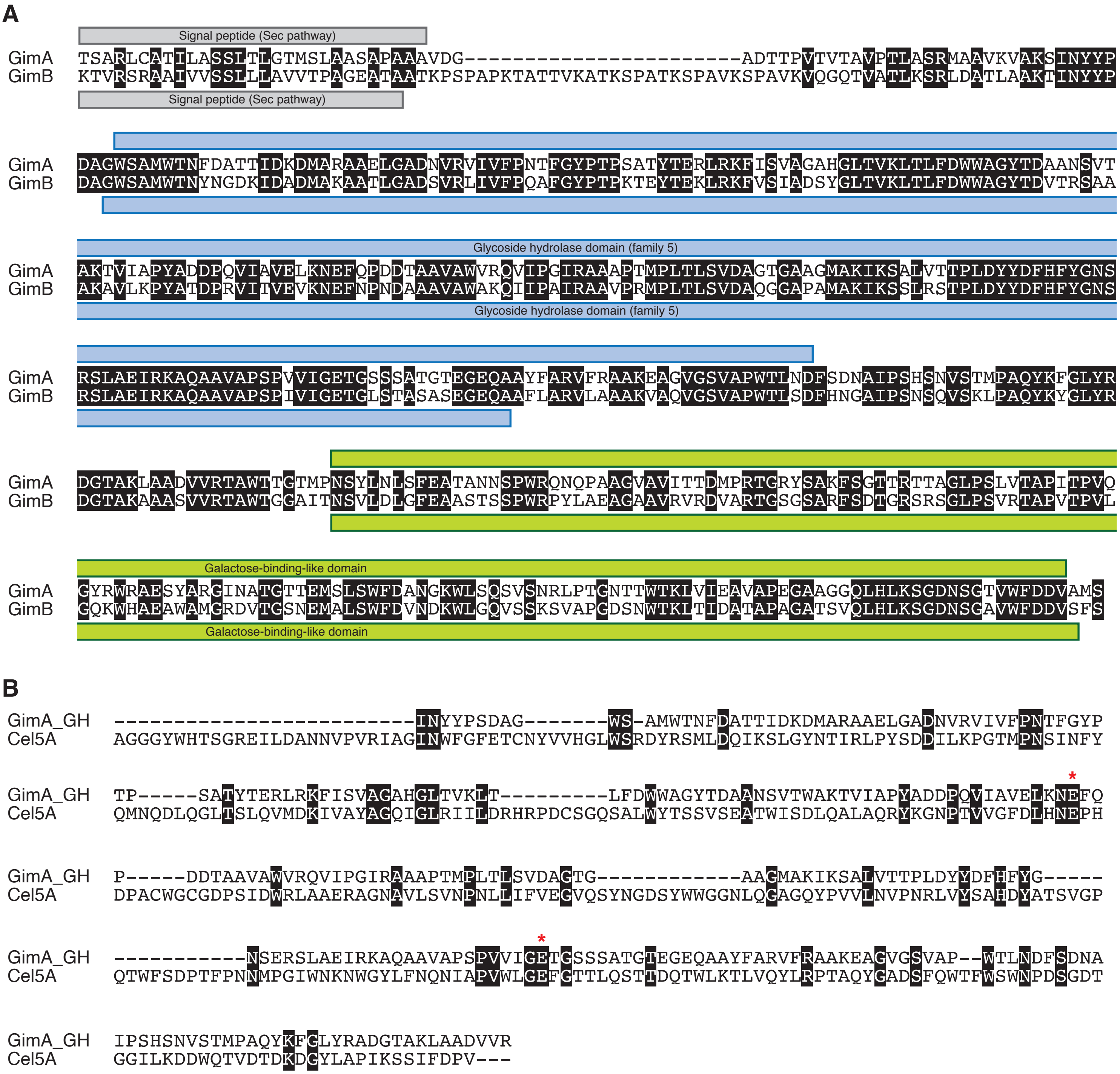

Fig. S1. Amino acid sequence alignment. (A) Sequence alignment of GimA and GimB. Both sequences show 62% identity. (B) Sequence alignment of the glycoside hydrolase domains of GimA (GimA_GH; residues 60-338) and endocellulase E1 from *A. cellulolyticus* (Cel5A). Two catalytic residues of Cel5A are shown with red asterisks, which correspond to Glu_174 and Glu_261 of GimA.

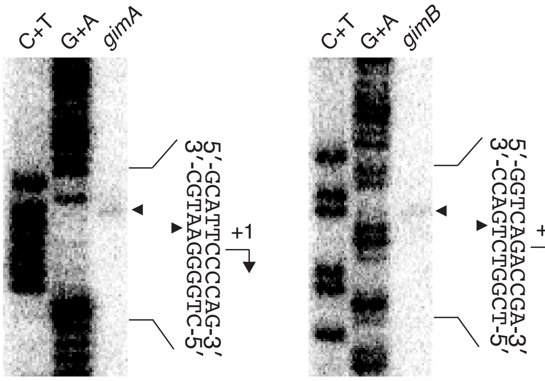

Fig. S2. High-resolution S1 nuclease mapping to analyze the transcriptional start points of *gimA* and *gimB*. The S1 nuclease-digested fragments are flanked by Maxam-Gilbert sequence ladders (C+T, G+A). Arrowheads indicate the positions of the S1 nuclease-protected fragments. The 5′ termini of the mRNAs were assigned to the positions indicated by the bent arrows because the fragments generated by the chemical sequencing reaction migrate 1.5 nucleotides ahead of the corresponding fragments generated by S1 nuclease digestion of the DNA-RNA hybrids.

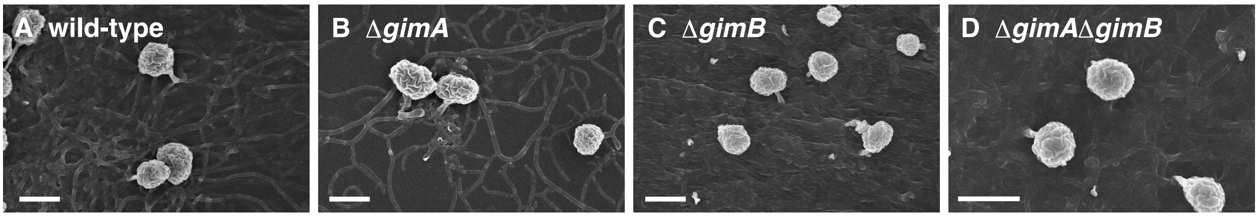

Fig. S3. Observation of mycelia and sporangia by scanning electron microscopy. Each strain was cultivated on HAT agar at 30°C for 7 days. Bars, 5 μm. (A) Wild-type strain. (B) Δ*gimA* strain. (C) Δ*gimB* strain. (D) Δ*gimA*Δ*gimB* strain.

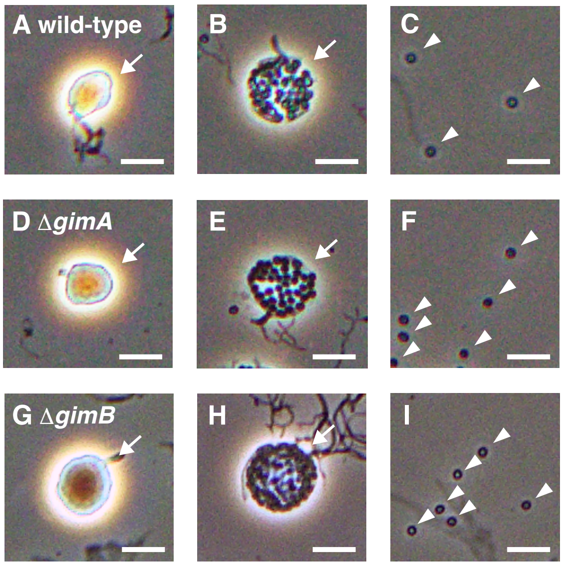

Fig. S4. Observation of sporangium dehiscence by phase-contrast microscopy. Sporangia produced on HAT agar were harvested and suspended into 25 mM histidine solution to induce sporangium dehiscence. Micrographs of the wild-type (A–C), Δ*gimA* (D–F), and Δ*gimB* (G–I) strains are shown. Panels A, D, and G show images taken immediately after suspension. Panels B, E, and H show images taken 15 min after suspension. Panels C, F, and I show images taken 30 min after suspension. Immediately after suspension, sporangia appeared phase-bright (A, D, and G). The sporangium envelope gradually became transparent before the release of spores (B, E, and H). Sporangia and released spores are indicated by arrows and arrowheads, respectively. Bars, 5 μm.

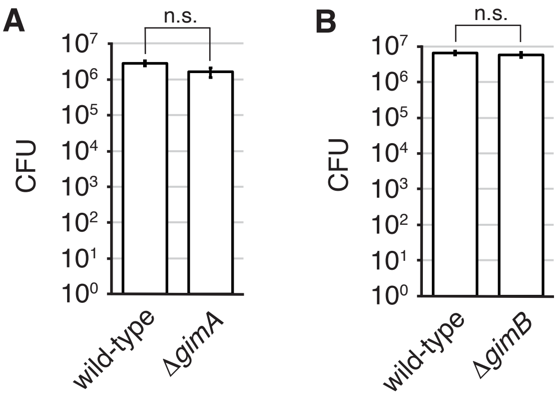

Fig. S5. Number of spores released from sporangia of the wild-type and Δ*gimA* strains (A), and of the wild-type and Δ*gimB* strains (B). Each strain was cultivated on HAT agar at 30°C for 7 days. Zoospores released from sporangia by pouring 25 mM NH_4HCO_3 solution on the sporangium-forming agar were counted as colony-forming units (CFUs) on YBNM agar. The values represent the mean ± standard error of three biological replicates. Differences are analyzed by the Student's *t*-test and "n.s." means no significant difference ($p = 0.07$ and 0.31 in panels A and B, respectively).

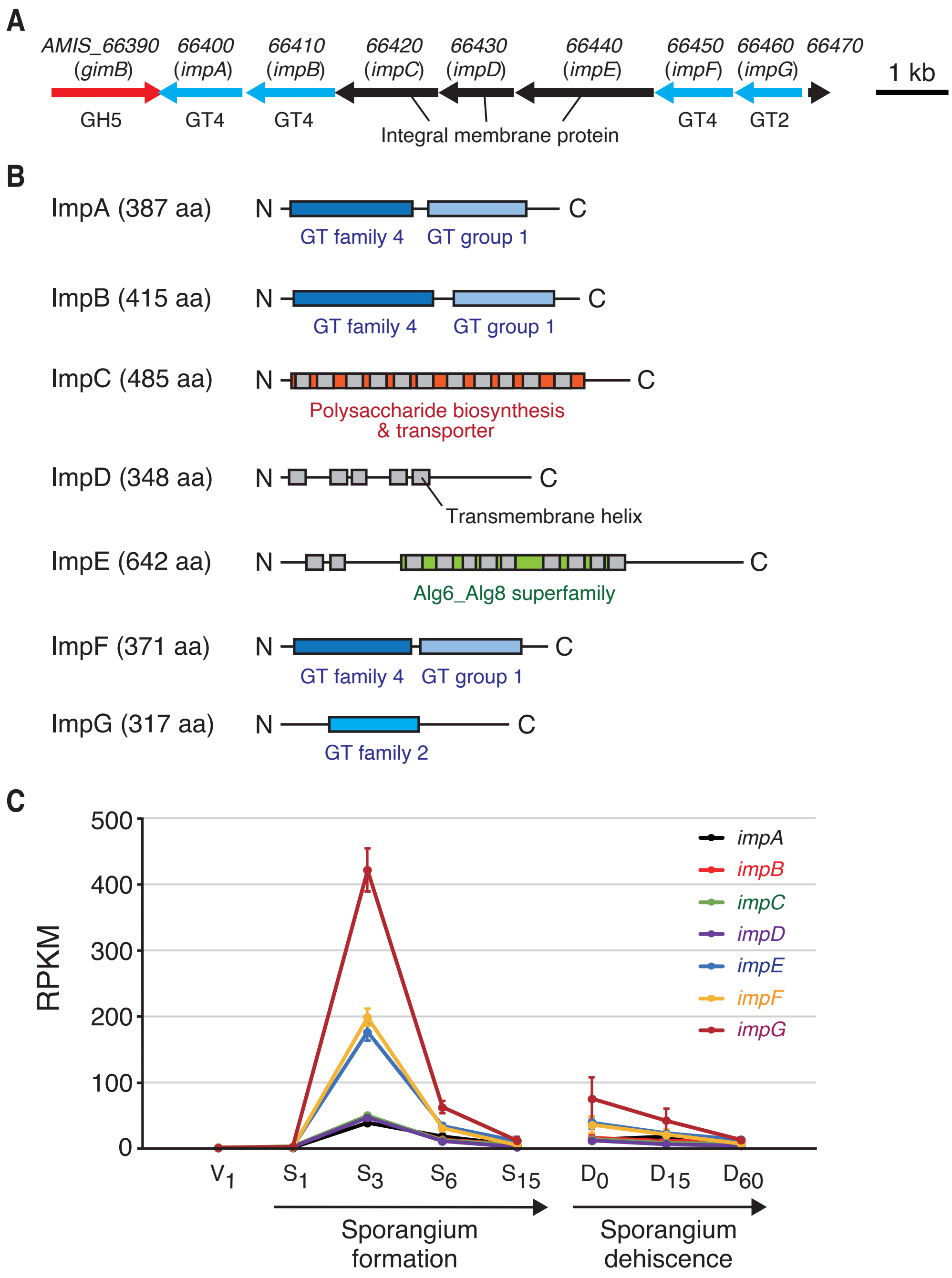

Fig. S6. Domain organization of gene products and transcriptional profiles of the *imp* gene cluster. (A) Gene organization of the *imp* gene cluster. Arrows indicate the locations of the open reading frames, including their length and direction. Gene identification numbers are shown above the arrows. Gene names are shown in parentheses. (B) Domain organization of gene products of the *imp* gene cluster. (C) Transcript levels of the seven genes comprising the *imp* gene cluster. Transcripts were examined using RNA-Seq analysis under various culture conditions. RNA samples were prepared from substrate hyphae grown on YBNM agar for 1 day (V_1), substrate hyphae or mixtures of substrate hyphae and sporangia grown on HAT agar for 1, 3, 6, and 15 days (S_1, S_3, S_6, and S_{15}, respectively), and sporangia (including some substrate hyphae) incubated in 25 mM histidine solution to induce sporangium dehiscence for 0, 15, and 60 min (D_0, D_{15}, and D_{60}, respectively). The average number of reads per kilobase of coding sequence per million mapped reads (RPKM) values ± standard errors from three biological replicates are shown.

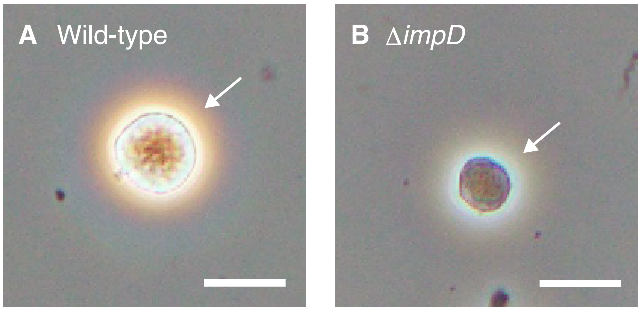

Fig. S7. Observation of sporangia of the wild-type (A) and Δ*impD* (B) strains using phase-contrast microscopy. Sporangia produced on HAT agar were harvested and suspended in water. Micrographs were taken immediately after suspension. Sporangia are indicated by arrows. Bars, 10 μm.

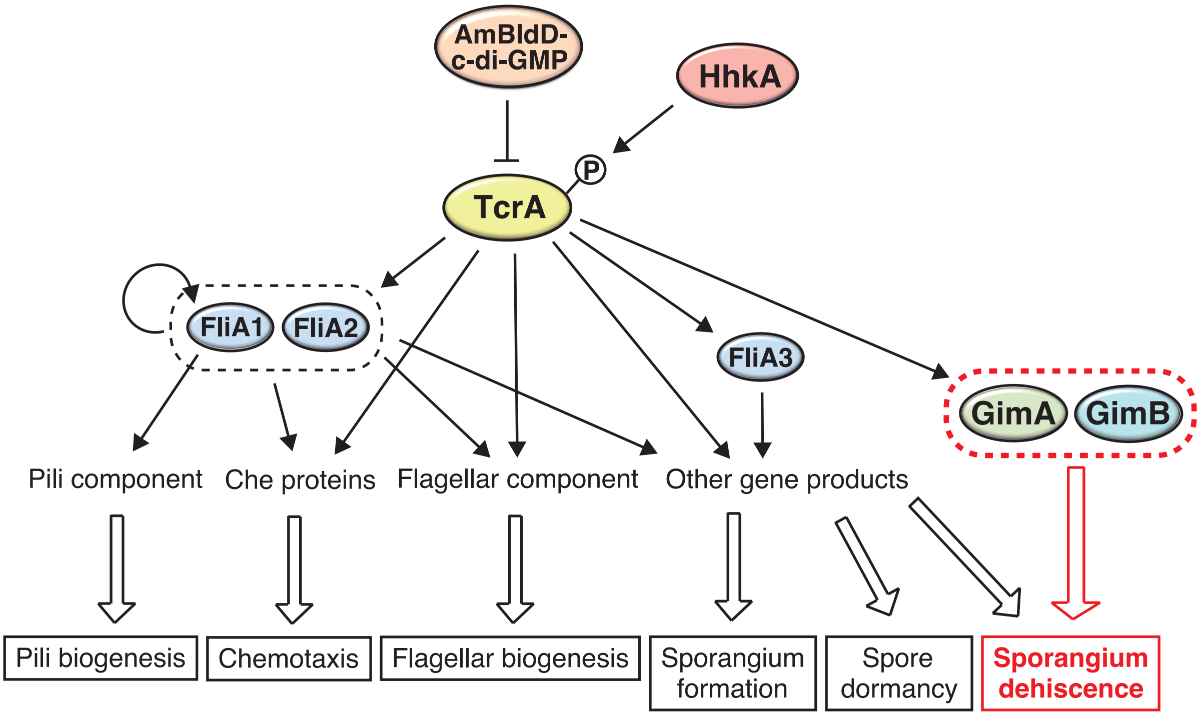

Fig. S8. TcrA-governed regulatory network of gene expression for morphological development in *A. missouriensis*. Arrows indicate positive controls, including direct and indirect regulations. Open arrows indicate the involvement of gene products in the biological phenomena described in boxes. The global transcriptional regulator TcrA activates target genes partly through alternative sigma factors FliA1, FliA2, and FliA3. The transcriptional regulator AmBldD in complex with tetrameric cyclic-di-GMPs represses *tcrA* transcription. The hybrid sensor histidine kinase HhkA has been proposed to be involved in the phosphorylation of the response regulator TcrA.

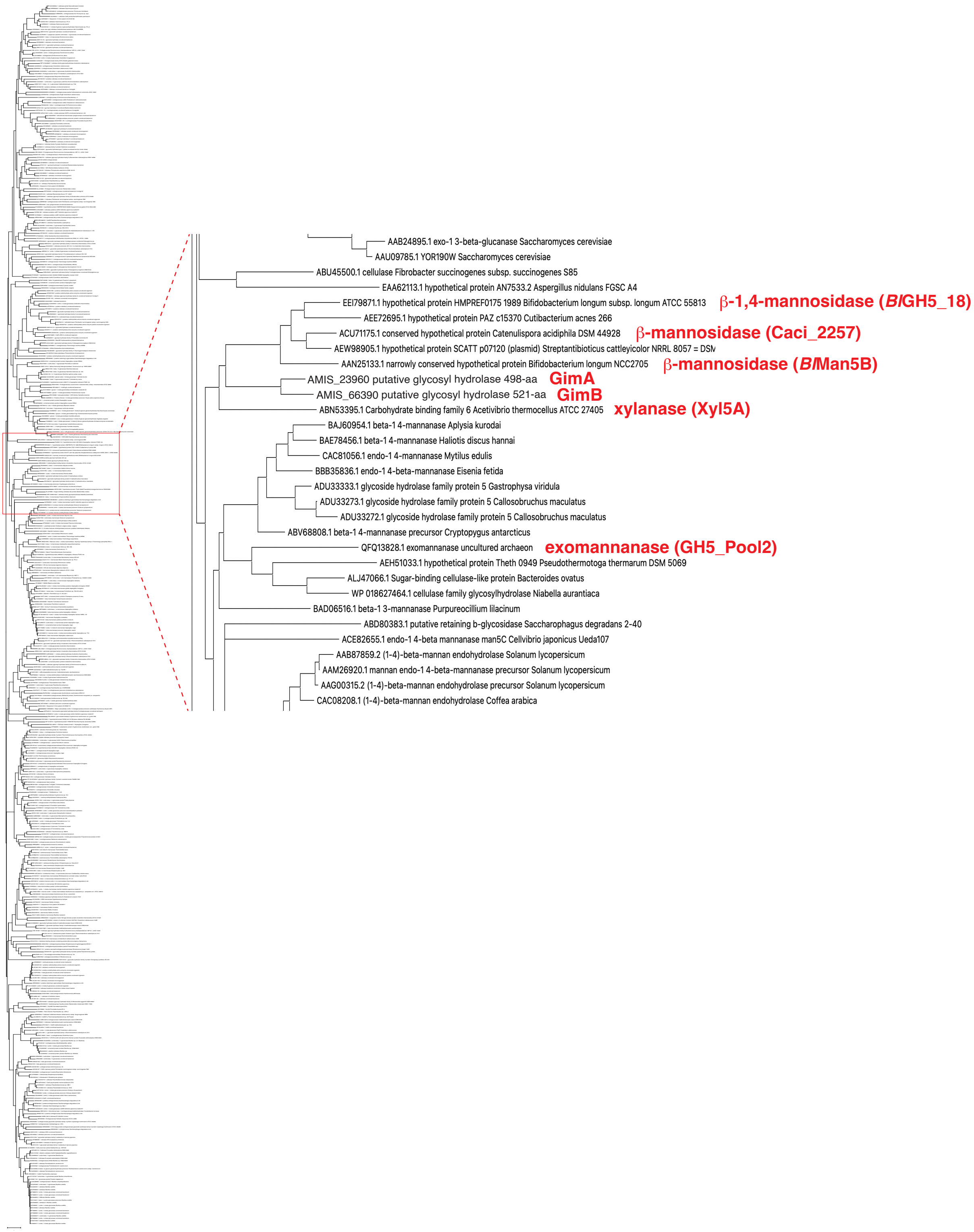

Fig. S9. Phylogenetic relationships of 467 glycoside hydrolases from family 5. A maximum likelihood tree was constructed using MEGA X (Kumar *et al*., 2018). A magnified description of the boxed area is shown on the right side of the tree. GimA, GimB, and several characterized enzymes are shown in red.

A

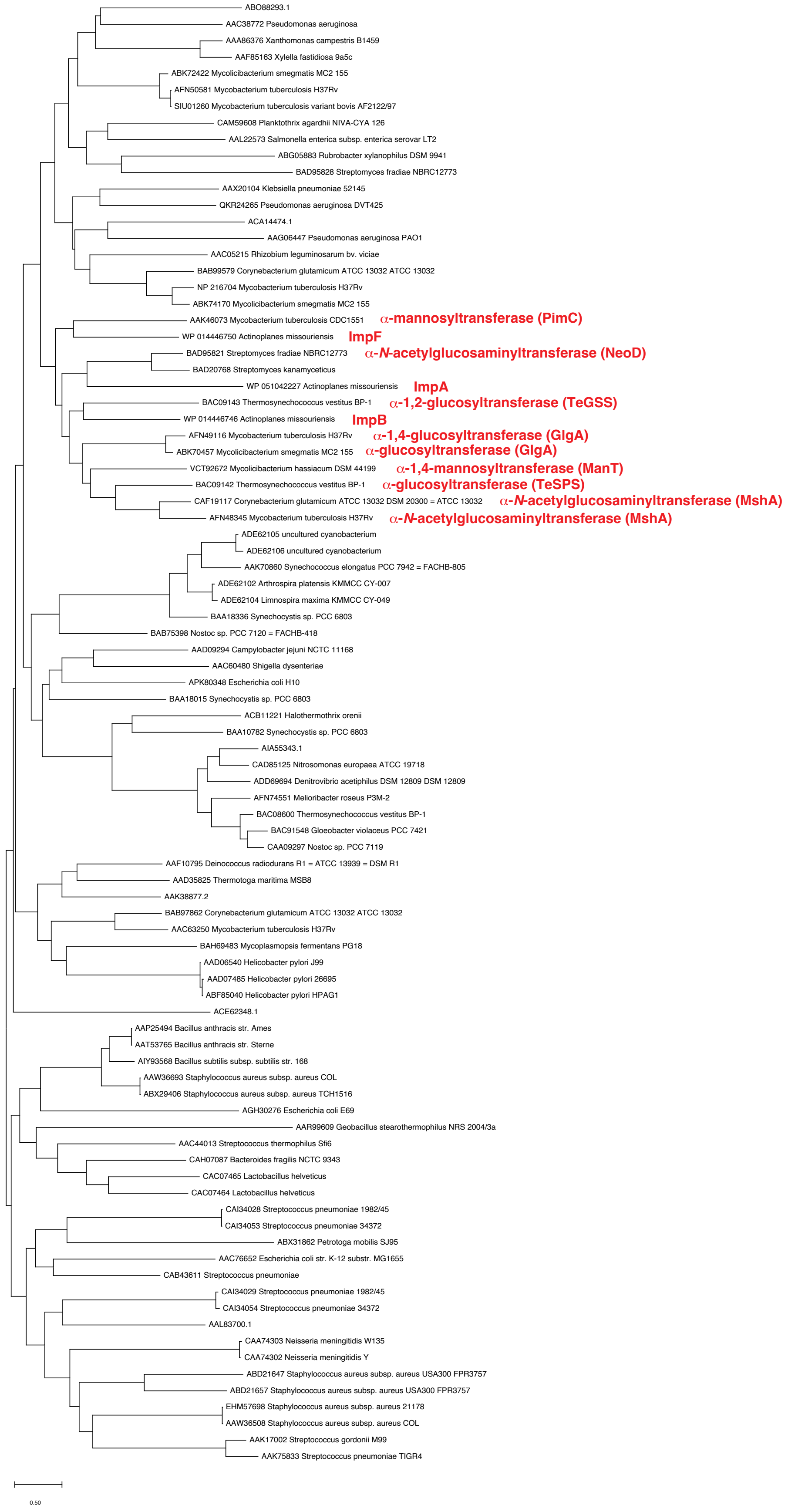
ABO88293.1
AAC38772 Pseudomonas aeruginosa
AAA86376 Xanthomonas campestris B1459
AAF85163 Xylella fastidiosa 9a5c
ABK72422 Mycolicibacterium smegmatis MC2 155
AFN50581 Mycobacterium tuberculosis H37Rv
SIU01260 Mycobacterium tuberculosis variant bovis AF2122/97
CAM59608 Planktothrix agardhii NIVA-CYA 126
AAL22573 Salmonella enterica subsp. enterica serovar LT2
ABG05883 Rubrobacter xylanophilus DSM 9941
BAD95828 Streptomyces fradiae NBRC12773
AAX20104 Klebsiella pneumoniae 52145
QKR24265 Pseudomonas aeruginosa DVT425
ACA14474.1
AAG06447 Pseudomonas aeruginosa PAO1
AAC05215 Rhizobium leguminosarum bv. viciae
BAB99579 Corynebacterium glutamicum ATCC 13032 ATCC 13032
NP 216704 Mycobacterium tuberculosis H37Rv
ABK74170 Mycolicibacterium smegmatis MC2 155
AAK46073 Mycobacterium tuberculosis CDC1551
α-mannosyltransferase (PimC)
WP 014446750 Actinoplanes missouriensis
ImpF
BAD95821 Streptomyces fradiae NBRC12773
α-N-acetylglucosaminyltransferase (NeoD)
BAD20768 Streptomyces kanamyceticus
WP 051042227 Actinoplanes missouriensis
ImpA
BAC09143 Thermosynechococcus vestitus BP-1
α-1,2-glucosyltransferase (TeGSS)
WP 014446746 Actinoplanes missouriensis
ImpB
AFN49116 Mycobacterium tuberculosis H37Rv
α-1,4-glucosyltransferase (GlgA)
ABK70457 Mycolicibacterium smegmatis MC2 155
α-glucosyltransferase (GlgA)
VCT92672 Mycolicibacterium hassiacum DSM 44199
α-1,4-mannosyltransferase (ManT)
BAC09142 Thermosynechococcus vestitus BP-1
α-glucosyltransferase (TeSPS)
CAF19117 Corynebacterium glutamicum ATCC 13032 DSM 20300 = ATCC 13032
α-N-acetylglucosaminyltransferase (MshA)
AFN48345 Mycobacterium tuberculosis H37Rv
α-N-acetylglucosaminyltransferase (MshA)
ADE62105 uncultured cyanobacterium
ADE62106 uncultured cyanobacterium
AAK70860 Synechococcus elongatus PCC 7942 = FACHB-805
ADE62102 Arthrospira platensis KMMCC CY-007
ADE62104 Limnospira maxima KMMCC CY-049
BAA18336 Synechocystis sp. PCC 6803
BAB75398 Nostoc sp. PCC 7120 = FACHB-418
AAD09294 Campylobacter jejuni NCTC 11168
AAC60480 Shigella dysenteriae
APK80348 Escherichia coli H10
BAA18015 Synechocystis sp. PCC 6803
ACB11221 Halothermothrix orenii
BAA10782 Synechocystis sp. PCC 6803
AIA55343.1
CAD85125 Nitrosomonas europaea ATCC 19718
ADD69694 Denitrovibrio acetiphilus DSM 12809 DSM 12809
AFN74551 Melioribacter roseus P3M-2
BAC08600 Thermosynechococcus vestitus BP-1
BAC91548 Gloeobacter violaceus PCC 7421
CAA09297 Nostoc sp. PCC 7119
AAF10795 Deinococcus radiodurans R1 = ATCC 13939 = DSM R1
AAD35825 Thermotoga maritima MSB8
AAK38877.2
BAB97862 Corynebacterium glutamicum ATCC 13032 ATCC 13032
AAC63250 Mycobacterium tuberculosis H37Rv
BAH69483 Mycoplasmopsis fermentans PG18
AAD06540 Helicobacter pylori J99
AAD07485 Helicobacter pylori 26695
ABF85040 Helicobacter pylori HPAG1
ACE62348.1
AAP25494 Bacillus anthracis str. Ames
AAT53765 Bacillus anthracis str. Sterne
AIY93568 Bacillus subtilis subsp. subtilis str. 168
AAW36693 Staphylococcus aureus subsp. aureus COL
ABX29406 Staphylococcus aureus subsp. aureus TCH1516
AGH30276 Escherichia coli E69
AAR99609 Geobacillus stearothermophilus NRS 2004/3a
AAC44013 Streptococcus thermophilus Sfi6
CAH07087 Bacteroides fragilis NCTC 9343
CAC07465 Lactobacillus helveticus
CAC07464 Lactobacillus helveticus
CAI34028 Streptococcus pneumoniae 1982/45
CAI34053 Streptococcus pneumoniae 34372
ABX31862 Petrotoga mobilis SJ95
AAC76652 Escherichia coli str. K-12 substr. MG1655
CAB43611 Streptococcus pneumoniae
CAI34029 Streptococcus pneumoniae 1982/45
CAI34054 Streptococcus pneumoniae 34372
AAL83700.1
CAA74303 Neisseria meningitidis W135
CAA74302 Neisseria meningitidis Y
ABD21647 Staphylococcus aureus subsp. aureus USA300 FPR3757
ABD21657 Staphylococcus aureus subsp. aureus USA300 FPR3757
EHM57698 Staphylococcus aureus subsp. aureus 21178
AAW36508 Staphylococcus aureus subsp. aureus COL
AAK17002 Streptococcus gordonii M99
AAK75833 Streptococcus pneumoniae TIGR4
0.50

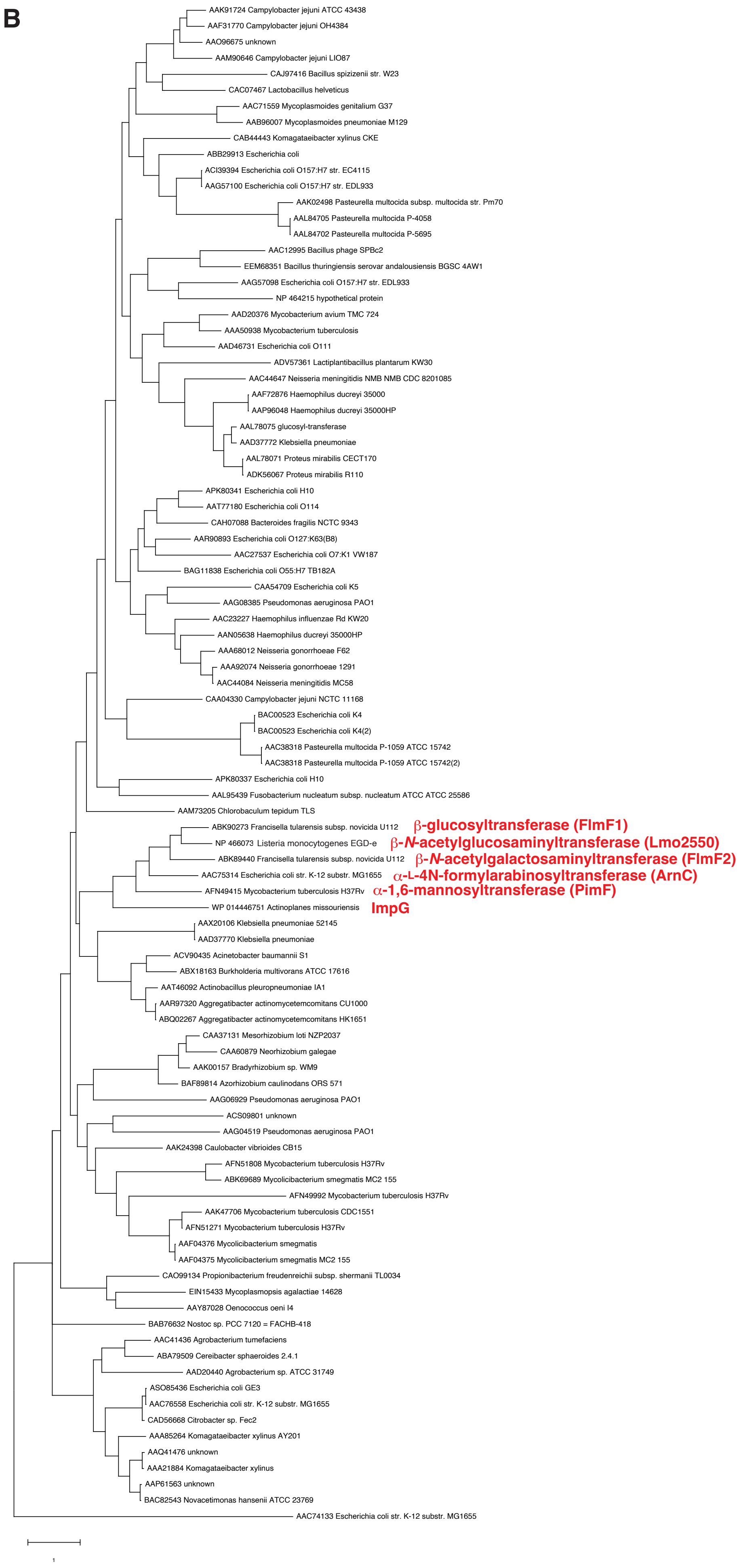

Fig. S10. Phylogenetic analysis of glycosyltransferases. Maximum likelihood trees of 89 glycosyltransferases from the GT4 family (A) and 95 glycosyltransferases from the GT2 family (B) were constructed using MEGA X. ImpA, ImpB, ImpF, ImpG and several characterized enzymes are shown in red.

Table S1. Primers used in this study

Primer name	Sequence (5' to 3')[a]	Used for
AMIS23960-UF1	GGAATTCGTGACCTGTTCGCCTGTGAG	Gene disruption
AMIS23960-UR1	GCTCTAGACGATGCCGCCAGGGACATCGT	Gene disruption
AMIS23960-DF1	CGTCTAGAACGTGGACGAAGCTGGTGAT	Gene disruption
AMIS23960-DR1	CGGAAGCTTGGATGACCGTCGGTGAGCTG	Gene disruption
AMIS66390-UF1	GGAATTCATCCTCTGCGACGCGGAGAT	Gene disruption
AMIS66390-UR1	GCTCTAGACGTCACGACGGCAAGGAGCA	Gene disruption
AMIS66390-DF1	GCTCTAGAACAAGCGTCCAGCTTCACCT	Gene disruption
AMIS66390-DR1	CGGAAGCTTCACGACTGGCTGATGTACAG	Gene disruption
AMIS66400-UF1	GGAATTCACCGGCCATCATCTGATCAA	Gene disruption
AMIS66400-UR1	GCTCTAGAGTGTCTCGAGGTCAGCGCGTT	Gene disruption
AMIS66400-DF1	GCTCTAGAGACGCCTACGACCGTTTGAT	Gene disruption
AMIS66400-DR1	CGGAAGCTTCCACGATTTCTGCACGCTCT	Gene disruption
AMIS66410-UF1	GGAATTCGTGCTGCTGGCGGTCGTCAT	Gene disruption
AMIS66410-UR1	CGTCTAGACCCGTTGATCTCCTCGTCGT	Gene disruption
AMIS66410-DF1	CGTCTAGAGTGGTTCCCGAGATCGAGGA	Gene disruption
AMIS66410-DR1	CGGAAGCTTCATGGCGAAGATCAAGTCGA	Gene disruption
AMIS66420-UF1	GGAATTCGGACCAGCAGCTATCTCGAT	Gene disruption
AMIS66420-UR1	CGTCTAGATTGACCACCGTGGTGGCCAT	Gene disruption
AMIS66420-DF1	CGTCTAGATCTCGAAGGTGAGAAGGACC	Gene disruption
AMIS66420-DR1	CGGAAGCTTCCACGATCGTCAGGTGGTAT	Gene disruption
AMIS66430-UF1	GGAATTCTCTACACCGCCGTGACAAGC	Gene disruption
AMIS66430-UR1	GCTCTAGAAGGAGCGCCACCAGGCAGAAC	Gene disruption
AMIS66430-DF1	GCTCTAGAGTCGACCAGCACCGGGTTCTAC	Gene disruption
AMIS66430-DR1	CCCAAGCTTATGGCGGTGAGCGTGTCGATC	Gene disruption
AMIS66440-UF1	GGAATTCTCAACGAGAACCGAGCCATC	Gene disruption
AMIS66440-UR1	GCTCTAGAGCCCAGAAGAAGGCGAACTG	Gene disruption
AMIS66440-DF1	GCTCTAGACACCAAGTTCGACACCATGC	Gene disruption
AMIS66440-DR1	CCCAAGCTTCTGAACATGCCGAGAATCGC	Gene disruption
AMIS66450-UF1	GGAATTCTTCCACGAGCCGATGGAGTC	Gene disruption
AMIS66450-UR1	CGTCTAGAGGTGATCACCTCGACGTCGTC	Gene disruption
AMIS66450-DF1	CGTCTAGAGTCTGGCTGCGATACGCCGATC	Gene disruption
AMIS66450-DR1	CCCAAGCTTATCGTCGTCATCGCACGCTC	Gene disruption
AMIS66460-UF1	GGAATTCACAGCCTGCGGATGGAACAC	Gene disruption
AMIS66460-UR1	GCTCTAGATGGTGGTCTCGGTTCCGGATG	Gene disruption
AMIS66460-DF1	GCTCTAGATGAGCAACCTCAACGCGCTC	Gene disruption

AMIS66460-DR1	CCCAAGCTTTGCAGGTGATCGCGATCAGG	Gene disruption
AMIS66400-460-UF1	GGAATTCGACAGCCTGCGGATGGAACA	Gene disruption
AMIS66400-460-UR1	GCTCTAGATGGCTCGGTTCTCGTTGACT	Gene disruption
AMIS66400-460-DF1	GCTCTAGAGACCGTTTGATCGCCCGGAT	Gene disruption
AMIS66400-460-DR1	CCCAAGCTTTCGGTGATCGAGAAACGACT	Gene disruption
AMIS23960-F1	GGAATTCGGGCCACTGATCAGCGACAA	Gene complementation
AMIS23960-R1	CGGAAGCTTCTGGTCACCACCTGACACCA	Gene complementation
AMIS66390-F1	GGAATTCGGGTACGGGCCTTACGTGGA	Gene complementation
AMIS66390-R1	CGGAAGCTTATCACGTTCGTGAACCGGTA	Gene complementation
AMIS5940-qF1	CGAGGAGTGCAAGGAGAAGG	qRT-PCR
AMIS5940-qR1	ATCGGGAAGTCACCCATGAA	qRT-PCR
AMIS23960-qF1	GCCCAGTACAAGTTCGGTCTC	qRT-PCR
AMIS23960-qR1	AGGTAGCTGTTCGGCATGGT	qRT-PCR
AMIS66390-qF1	GACGAATTACAACGGCGACA	qRT-PCR
AMIS66390-qR1	GGAACACGATGAGACGGACA	qRT-PCR
AMIS23960-S1-F1	CGAATTCAGTTCCAGGAGACCCAGTAC	S1 nuclease mapping
AMIS23960-S1-R1	GGAGGAAGCCAGAATGGTGG	S1 nuclease mapping
AMIS66390-S1-F1	CGAATTCACGTACCCGGCGGAGTGACG	S1 nuclease mapping
AMIS66390-S1-R1	AGGATACGACGATCGCTGCG	S1 nuclease mapping
AMIS23960-EMSA-F1	GAGTTCCAGGAGACCCAGTA	EMSA
AMIS23960-EMSA-R1	GTGAGGGAGGAAGCCAGAAT	EMSA
AMIS66390-EMSA-F1	GTCTACACCCCGAGGACGTA	EMSA
AMIS66390-EMSA-R1	TGCGAACAGTCTTCACGGGA	EMSA
AMIS23960-ORF-F1	GGAATTCCATATGGCATCGGCGCCGGCGGCGGCCGTGGA	Recombinant protein production
AMIS23960-ORF-R1	CCCAAGCTTTCAGGACATGGCTACGTCGT	Recombinant protein production
AMIS23960-ORF-F2	GCTGAAGAACCAGTTCCAGCC	Recombinant protein production
AMIS23960-ORF-F3	TGGTGATCGGCCAGACCGGGTCC	Recombinant protein production
AMIS23960-ORF-R3	GGCTGGAACTGGTTCTTCAGC	Recombinant protein production
AMIS23960-ORF-R4	GGACCCGGTCTGGCCGATCACCA	Recombinant protein production
AMIS66390-ORF-F1	GGAATTCCATATGGCGACCAAGCCGAGCCCGGCCCCGAA	Recombinant protein production
AMIS66390-ORF-R2	CCCAAGCTTGGAGAACGACACGTCGTCGA	Recombinant protein production

[a] The recognition sequences for restriction enzymes are underlined.

Movie S1 (separate file). Induction of spore release from Δ*gimA*Δ*gimB* sporangia by His-GimA protein. The sporangia and mycelia of the Δ*gimA*Δ*gimB* strain were incubated in 25 mM histidine solution at room temperature for 1 h. The recombinant His-GimA protein was exogenously added to the suspension containing the sporangia with transparent sporangium envelope. Immediately after His-GimA addition, the suspension was observed by phase-contrast microscopy.

Supplemental reference

Kumar S, Stecher G, Li M, Knyaz C, Tamura K. 2018. MEGA X: Molecular evolutionary genetics analysis across computing platforms. Mol Biol Evol 35:1547-1549.
